# Pharmacological Interventions to Treat Antipsychotic-Induced Dyslipidemia in Schizophrenia Patients: A Systematic Review and Meta Analysis

**DOI:** 10.3389/fpsyt.2021.642403

**Published:** 2021-03-17

**Authors:** Pruntha Kanagasundaram, Jiwon Lee, Femin Prasad, Kenya A. Costa-Dookhan, Laurie Hamel, Madeleine Gordon, Gary Remington, Margaret K. Hahn, Sri Mahavir Agarwal

**Affiliations:** ^1^Centre for Addiction and Mental Health, Toronto, ON, Canada; ^2^School of Pharmacy, University of Waterloo, Waterloo, ON, Canada; ^3^Temerty Faculty of Medicine, Institute of Medical Science, University of Toronto, Toronto, ON, Canada; ^4^Sunnybrook Health Sciences Centre, Toronto, ON, Canada; ^5^Department of Psychiatry, Temerty Faculty of Medicine, University of Toronto, Toronto, ON, Canada; ^6^Banting and Best Diabetes Centre, University of Toronto, Toronto, ON, Canada

**Keywords:** schizophrenia, antipsychotics, dyslipidemia, systematic review, meta-analysis

## Abstract

**Introduction:** Antipsychotic-induced dyslipidemia represents a common adverse effect faced by patients with schizophrenia that increases risk for developing further metabolic complications and cardiovascular disease. Despite its burden, antipsychotic-induced dyslipidemia is often left untreated, and the effectiveness of pharmacological interventions for mitigating dyslipidemia has not been well-addressed. This review aims to assess the effectiveness of pharmacological interventions in alleviating dyslipidemia in patients with schizophrenia.

**Methods:** Medline, PsychInfo, and EMBASE were searched for all relevant English articles from 1950 to November 2020. Randomized placebo-controlled trials were included. Differences in changes in triglycerides, HDL cholesterol, LDL cholesterol, and VLDL cholesterol levels between treatment and placebo groups were meta-analyzed as primary outcomes.

**Results:** Our review identified 48 randomized controlled trials that comprised a total of 3,128 patients and investigated 29 pharmacological interventions. Overall, pharmacological interventions were effective in lowering LDL cholesterol, triglycerides, and total cholesterol levels while increasing the levels of HDL cholesterol. Within the intervention subgroups, approved lipid-lowering agents did not reduce lipid parameters other than total cholesterol level, while antipsychotic switching and antipsychotic add-on interventions improved multiple lipid parameters, including triglycerides, LDL cholesterol, HDL cholesterol, and total cholesterol. Off label lipid lowering agents improved triglycerides and total cholesterol levels, with statistically significant changes seen with metformin.

**Conclusion:** Currently available lipid lowering agents may not work as well in patients with schizophrenia who are being treated with antipsychotics. Additionally, antipsychotic switching, antipsychotic add-ons, and certain off label interventions might be more effective in improving some but not all associated lipid parameters. Future studies should explore novel interventions for effectively managing antipsychotic-induced dyslipidemia.

**Registration:** PROSPERO 2020 CRD42020219982; https://www.crd.york.ac.uk/prospero/display_record.php?ID=CRD42020219982.

## Introduction

Dyslipidemia refers to abnormalities in lipid levels such as increases in total and low-density lipoprotein (LDL) cholesterols, low concentrations of high-density lipoprotein (HDL) cholesterols, and high triglyceride levels. This metabolic abnormality causes almost a third of ischemic heart disease and a fifth of global cerebrovascular disease (1). Patients with schizophrenia are at an increased risk of developing cardiovascular disease in part due to the illness itself (2–7), as well as a higher prevalence of well-known lifestyle factors that promote cardiovascular disease risk, namely sedentary lifestyle, poor diet, and smoking (8, 9). Antipsychotics are the cornerstone of treatment in schizophrenia and are widely prescribed across other psychiatric conditions (10). However, their use is associated with severe metabolic adverse effects, including weight gain, dyslipidemia, insulin resistance, and risk of type 2 diabetes mellitus (T2D) in a population burdened with premature cardiovascular mortality.

While the prevalence of dyslipidemia and consequent effects on morbidity and mortality are high worldwide among the general population, particular subgroups may be at a greater risk. In particular, patients with schizophrenia are at an increased risk of dyslipidemia and its associated influence on cardiovascular disease and metabolic dysfunction (11, 12). Despite its high prevalence and associated cardiovascular risk, dyslipidemia often goes untreated among patients with schizophrenia. Reported rates for non-treatment are almost 90% (13–15), and patients with schizophrenia are often medically underserved and disadvantaged in their physical health care (16–18). As shown by results from a study by the National Institute of Mental Health, namely the Recovery After an Initial Schizophrenia Episode–Early Treatment Program (RAISE-ETP), at baseline more than half of patients (161/394 or 56.5%) had dyslipidemia and only 0.5% were receiving treatment (16).

Previous discussions addressing antipsychotic-induced metabolic abnormalities in patients with schizophrenia have largely focused on weight gain or metabolic syndrome, and not dyslipidemia *per se* (19–21). Only a few studies have investigated approved lipid lowering agents for treating dyslipidemia in schizophrenia (22–30). More commonly, as reported in a 2014 review by Tse et al. a wide variety of pharmacological agents have been investigated to treat dyslipidemia in patients with schizophrenia, including treatment with omega-3 fatty acids, fluvoxamine, topiramate, metformin, sibutramine, telmisartan, ramelteon, and valsartan (31). Antipsychotic switching and adding aripiprazole have also been evaluated as strategies to improve lipid outcomes in patients with schizophrenia (32–38). Given the variety of approaches used to address dyslipidemia in this patient population, as well as the absence of clear clinical guidelines, it is important to summarize the available evidence and guide clinical decision making. Hence, we performed a systematic review and meta-analysis of randomized controlled trials to compare the effects of pharmacological interventions vs. placebo treatment in antipsychotic-induced dyslipidemia in patients with schizophrenia.

## Methods

The protocol for the review is registered on PROSPERO (PROSPERO 2020 CRD42020219982; https://www.crd.york.ac.uk/prospero/display_record.php?ID=CRD42020219982). PRISMA guidelines were used for study design and reporting.

### Search

We searched for studies published between 1950 and November 2020 using Medline, PsychInfo and EMBASE databases, with the following search string: *psychotic disorder* OR *schizophrenia* OR *schizoaffective* OR *schizophreniform* OR *psychosis* OR *first episode* AND *hyperlipidemia* OR *triglycerides* OR *cholesterol* OR *lipid* OR *LDL cholesterol* OR *VLDL cholesterol* OR *HDL cholesterol*. The search was limited to studies conducted in human participants and published in English. References cited in previously published literature reviews and meta-analyses pertaining to interventions for metabolic disturbances in the schizophrenia population were reviewed for additional studies.

### Inclusion Criteria

Articles were initially screened using title and abstract, based on the study's relevance to our meta-analysis. Thereafter, articles were further screened to ensure that studies met the following inclusion criteria: (a) randomized placebo-controlled trial; (b) diagnosis of schizophrenia spectrum disorders comprising the majority (>50%) of study populations; (c) patients with current metabolic abnormalities; (d) an active pharmacological intervention used to improve metabolic abnormalities or an antipsychotic switching/add-on method if the antipsychotic change is aimed to improve metabolic parameters; and (e) primary outcome listed as lipids or other metabolic measures if lipid outcomes were included in the list of metabolic measures.

Studies were excluded from analysis during the final screening stage if (a) not aimed at improving metabolic measures; (b) comparing different modes of antipsychotic administration (i.e., deltoid, sublingual, gluteal etc.); (c) comparing effectiveness between different antipsychotics; (d) evaluating non-pharmacological intervention (e.g., behavioral interventions, dietary modulations etc.); or, (e) evaluating strategies to prevent dyslipidemia (i.e., patients did not have metabolic abnormalities or dyslipidemia at baseline).

### Outcomes Extracted

The primary outcomes included the following lipid parameters: total cholesterol, triglycerides, LDL cholesterol, HDL cholesterol, and very low-density lipoprotein cholesterol (VLDL) cholesterol. Additional secondary outcome data were also extracted including body weight, body mass index (BMI), waist circumference, waist to hip ratio, fasting blood glucose, fasting insulin, hemoglobin A1c (HbA1c), diastolic blood pressure, systolic blood pressure, the homeostatic model assessment of insulin resistance (HOMA-IR), and total positive and negative symptom scale (total PANSS). Outcomes were extracted for both the intervention and placebo groups, where the placebo groups were used as comparators. Outcomes were extracted by two authors (PK and KC-D) and were checked by authors, FP and JL. For studies that examined multiple doses of the same intervention, the data pertaining to the higher dose were extracted.

### Data Analysis

Review Manager 5.4 (Revman 5.4.0 (Mac Version) Cochrane Collaboration, Oxford) was used to analyze the data extracted from the final list of included articles. Continuous outcomes were reported using mean differences (MD) with 95% confidence intervals (CIs), following the inverse variance statistical method and random effects model to account for study heterogeneity. Missing standard deviations (SDs) were calculated using other available statistics that were reported. Endpoint data were primarily used unless not available, in which case mean change data were used. Endpoint and change data were combined during the analysis, as we used mean difference rather than standardized mean difference (39). For Emsley et al. which was a double-blind trial with an open-label extension (27), data were extracted at the endpoint of the double-blind phase. Study heterogeneity was calculated using the *I*^2^ statistic, with significant heterogeneity being classified as *I*^2^ ≥ 50%. Significant statistical differences were classified as *p* < 0.05. Changes in lipid profiles (i.e., HDL cholesterol, LDL cholesterol, VLDL cholesterol, triglycerides, and total cholesterol) were assessed for all interventions pooled and for the following 4 subgroups: lipid lowering agents; antipsychotic switching or antipsychotic add-on interventions; the off-label lipid lowering agent metformin; and other off-label lipid lowering agents.

All included studies were judged for risk of bias in random sequence generation, allocation concealment, blinding of participants and personnel, blinding of outcome assessment, incomplete outcome data, selective reporting, and other bias using the Cochrane Risk of Bias tool (40). Studies were judged to have either a low, high, or unclear risk of bias. Sensitivity analyses were conducted after removing studies found to be at high risk of bias to examine their impact on findings.

## Results

**Figure 1:**
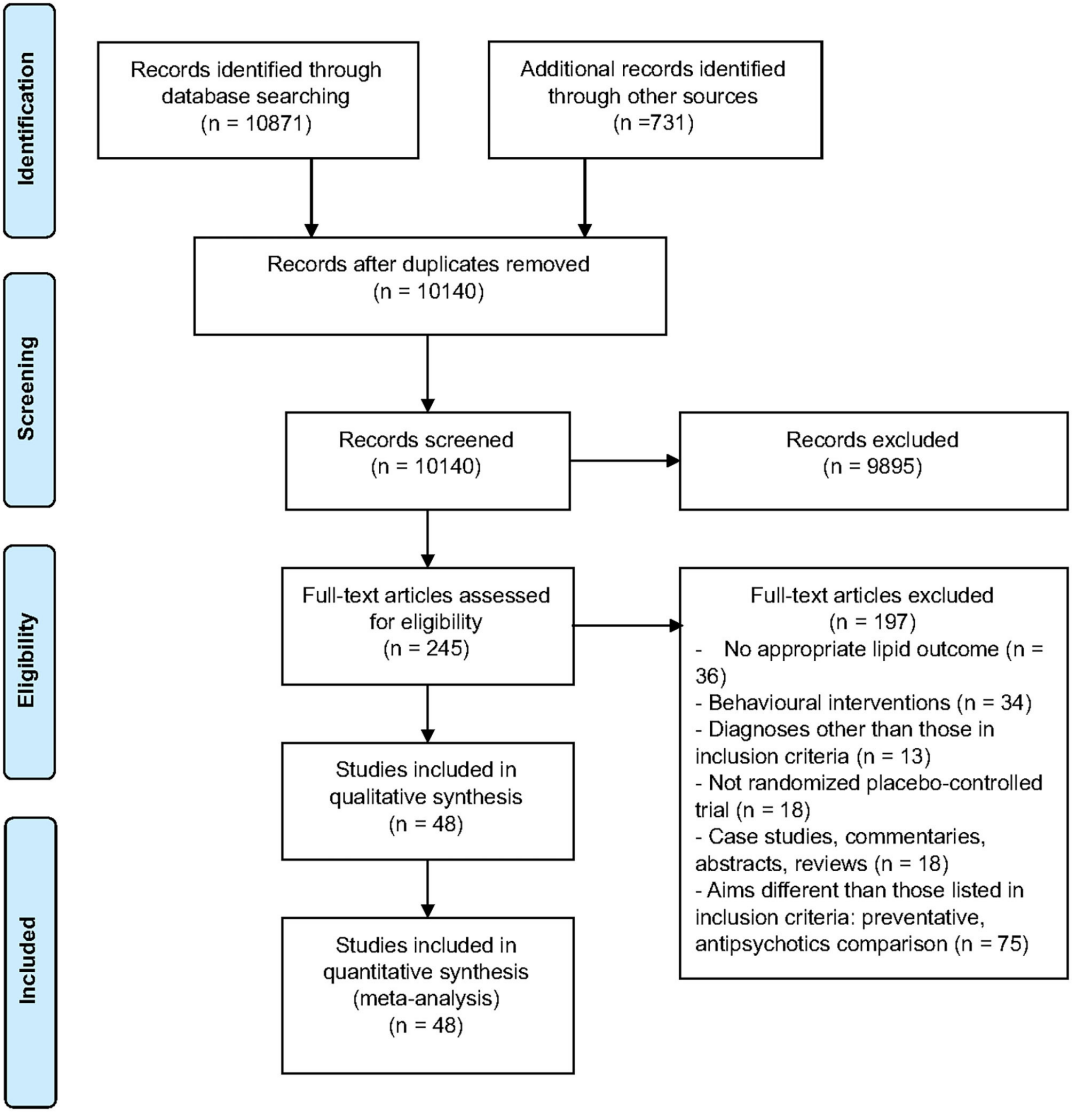
PRISMA flow chart describing the selection process of included articles for the systematic review and meta-analysis.

Of the 244 full-text articles screened, 48 articles (*n* = 3,128) met criteria for inclusion (Figure 1, Supplementary Table 1). Forty-three studies were double-blind, 2 were open-label, one had blinding but did not specify level, and the remaining 2 studies did not provide information on blinding. All studies included adult populations (18 years or older). The average age (±SD) of participants receiving interventions was 40.1 (±12.8) years, vs. 40.6 (±9.6) for those receiving placebo. A total of 74% of participants in both the intervention and placebo groups were male, with 89.2% diagnosed with schizophrenia. Trials were 4–24 weeks long, with a mean duration (±SD) of 13.1 (±5.7) weeks. Studies comprised a total of 29 interventions. Lipid lowering agents included omega-3 fatty acids [(26–28, 30), *N* = 4, *n* = 250] and pravastatin [(29), *N* = 1, *n* = 49]. Antipsychotic switching or add-on interventions included the following: switching to quetiapine [(41), *N* = 1, *n* = 133]; adding aripiprazole [(33, 34, 38), *N* = 3, *n* = 322]; and, switching to aripiprazole [(35–37), *N* = 3, *n* = 390]. Off label lipid modulating agents included the following: metformin [(42–47), *N* = 6, *n* = 565], reboxetine [(48), *N* = 1, *n* = 54], nizatidine [(49), *N* = 1, *n* = 54], atomoxetine [(50), *N* = 1, *n* = 29], combination of metformin and sibutramine [(51), *N* = 1, *n* = 28], rosiglitazone [(52, 53), *N* = 2, *n* = 47], ramelteon [(54), *N* = 1, *n* = 20], telmisartan [(55), *N* = 1, *n* = 43], vitamin D and probiotic combination [(56), *N* = 1, *n* = 60], sibutramine [(17, 57), *N* = 2, *n* = 55]; dehydroepiandrosterone [DHEA; (58), *N* = 1, *n* = 43], exenatide [(59), *N* = 1, *n* = 40], orlistat [(60), *N* = 1, *n* = 63], vitamin D [(61), *N* = 1, *n* = 47], liraglutide [(62), *N* = 1, *n* = 97], intranasal insulin [(63), *N* = 1, *n* = 39], minocycline [(64), *N* = 1, *n* = 55], fluvoxamine [(65, 66), *N* = 2, *n* = 153], naltrexone and bupropion combination [(67), *N* = 1, *n* = 21], melatonin [(68, 69), *N* = 2, *n* = 80], pioglitazone [(70), *N* = 1, *n* = 52], Liuyu decoction, traditional Chinese medicine [(71), *N* = 1, *n* = 154], a combination of celery, dill, and green tea [(72), *N* = 1, *n* = 60], naltrexone [(73, 74), *N* = 2, *n* = 47], Konjac powder [(75), *N* = 1, *n* = 59], and resveratrol [(76), *N* = 1, *n* = 19]. Baseline antipsychotic use by participants included olanzapine (*N* = 27), clozapine (*N* = 25), risperidone (*N* = 11), quetiapine (*N* = 8), aripiprazole (*N* = 5), ziprasidone (*N* = 2), paliperidone (*N* = 2), haloperidol (*N* = 1), fluphenazine (*N* = 1), flupenthixol (*N* = 1), clopenthixol (*N* = 1), and sulpiride (*N* = 1), chlorpromazine (*N* = 1), perphenazine (*N* = 1), zuclopenthixol (*N* = 1), chlorprothixene (*N* = 1), amisulpride (*N* = 1), sertindole (*N* = 1), and sulpiride (*N* = 1).

### Primary Outcomes: Lipid Profile

**Figure 2:**
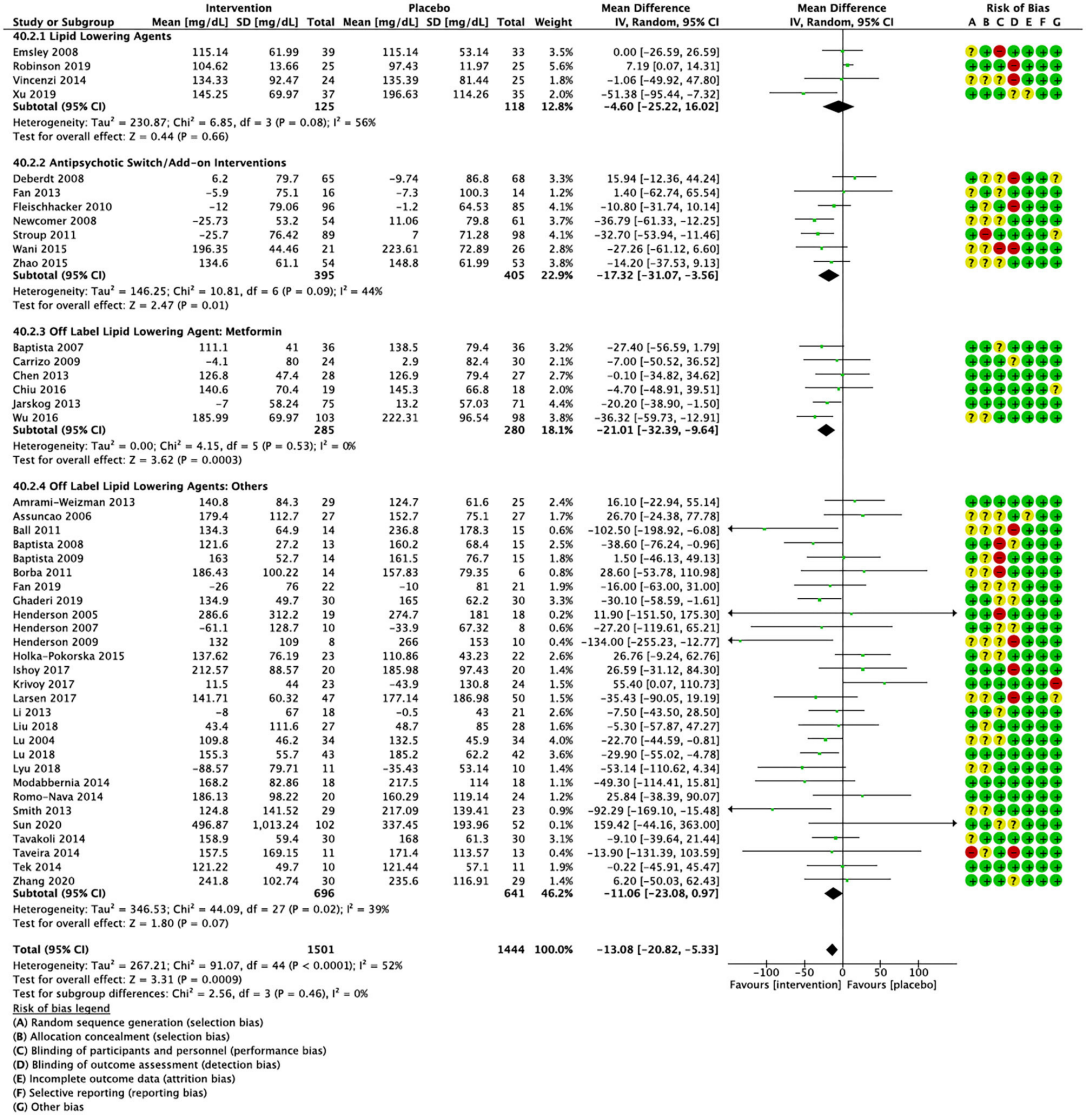
Forest plot showing the pooled mean difference in changes in triglycerides (mg/dL) for all interventions as compared to placebo along with subgroup analyses and Risk of Bias assessments.

**Figure 3:**
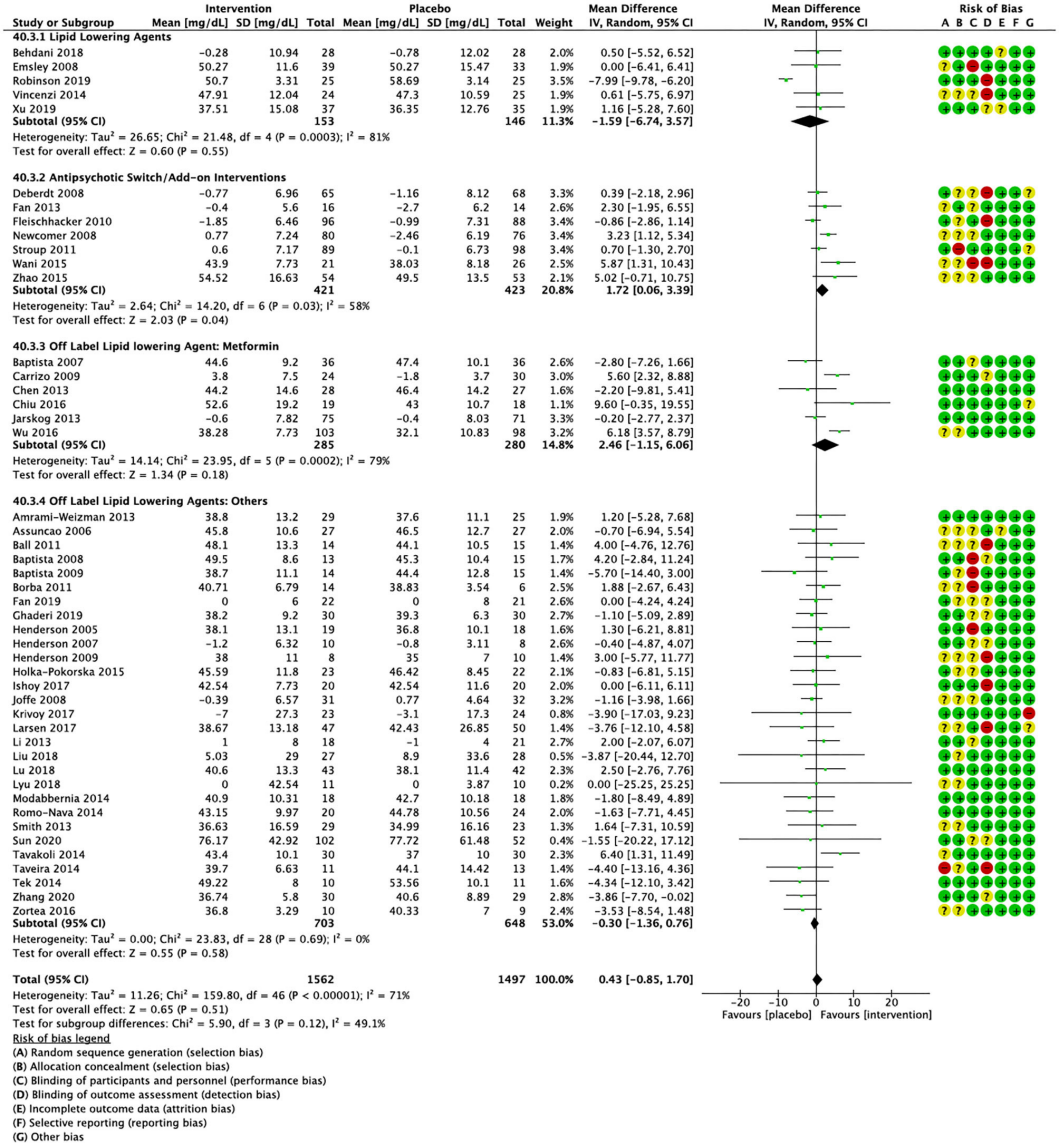
Forest plot showing the pooled mean difference in changes in high-density lipoprotein cholesterol (mg/dL) for all interventions as compared to placebo along with subgroup analyses and Risk of Bias assessments.

**Figure 4:**
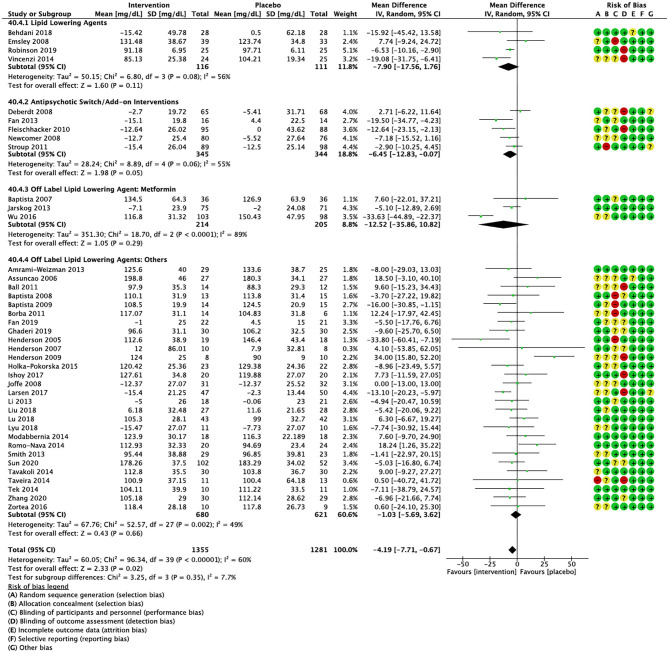
Forest plot showing the pooled mean difference in changes in low-density lipoprotein cholesterol (mg/dL) for all interventions as compared to placebo along with subgroup analyses and Risk of Bias assessments.

**Figure 5:**
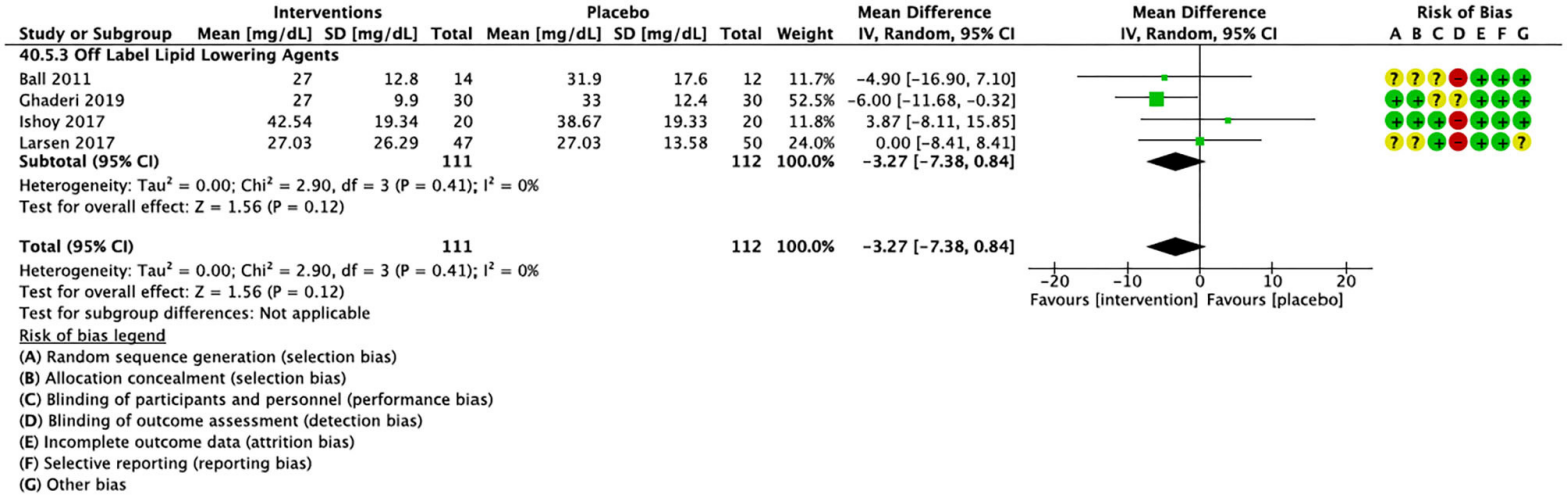
Forest plot showing the pooled mean difference in changes in very low-density lipoprotein cholesterol (mg/dL) for all interventions as compared to placebo and Risk of Bias assessments.

**Figure 6:**
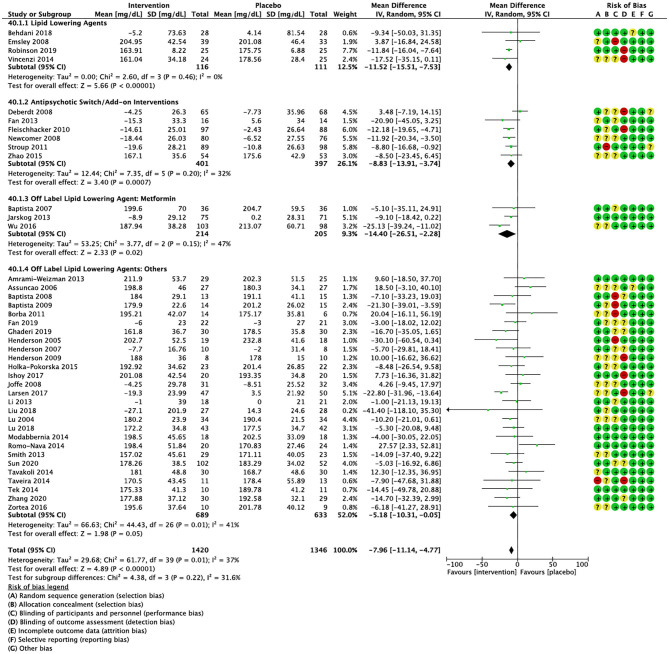
Forest plot showing the pooled mean difference in changes in total cholesterol (mg/dL) for all interventions as compared to placebo along with subgroup analyses and Risk of Bias assessments.

Compared to placebo, pharmacological interventions were associated with a pooled mean difference of −13.08 mg/dL (CI: −20.82, −5.33; *p* = 0.0009) for triglycerides (Figure 2), 0.43 mg/dL (CI: −0.85, 1.70; *p* = 0.51) for HDL (Figure 3), −4.19 mg/dL (CI: −7.71, −0.67; *p* = 0.02) for LDL cholesterol (Figure 4), −3.27 mg/dL (CI: −7.38, 0.84; *p* = 0.12) for VLDL cholesterol (Figure 5), and −7.96 mg/dL (CI: −11.14, −4.77; *p* < 0.00001) for total cholesterol (Figure 6). Heterogeneity was low to moderate for most outcomes: *I*^2^ = 71% for HDL, *I*^2^ = 60% for LDL cholesterol, *I*^2^ = 0% for VLDL cholesterol, *I*^2^ = 52% for triglycerides, *I*^2^ = 37% for total cholesterol.

#### Lipid Lowering Agents

Lipid lowering agents were associated with significant reductions in total cholesterol compared to placebo (Figure 6; *N* = 4, *n* = 227; WMD = −11.52 mg/dL, CI: −15.51, −7.53; *p* < 0.00001; *I*^2^ = 0). There were no significant differences in triglycerides (Figure 2; *N* = 4, *n* = 243; *I*^2^ = 56), HDL cholesterol (Figure 3; *N* = 5, *n* = 299; *I*^2^ = 81), and LDL cholesterol (Figure 4; *N* = 4, *n* = 227; *I*^2^ = 56) levels. None of the lipid lowering agent studies examined VLDL cholesterol.

#### Antipsychotic Switching/Add-on Interventions

Antipsychotic switch/add-on strategies were associated with significant decreases in triglycerides (Figure 2; *N* = 7, *n* = 800; WMD = −17.32 mg/dL, CI: −31.07, −3.56; *p* = 0.01; *I*^2^ = 44), LDL cholesterol (Figure 4; *N* = 5, *n* = 689; WMD = −6.45 mg/dL, CI: −12.83, −0.07; *p* = 0.05; *I*^2^ = 55), and total cholesterol levels (Figure 6; *N* = 6, *n* = 798; WMD = −8.83 mg/dL; CI: −13.91, −3.74; *p* = 0.0007; *I*^2^ = 32) in comparison to placebo. A significant increase was noted for HDL cholesterol level (Figure 3; *N* = 7, *n* = 844; WMD = 1.72 mg/dL; CI: 0.06, 3.39; *p* = 0.04; *I*^2^ = 58). None of the antipsychotic switching/add-on studies examined VLDL cholesterol.

#### Off Label Lipid Lowering Agent: Metformin

Metformin was associated with significant reductions in triglycerides (Figure 2; *N* = 6, *n* = 565; WMD = −21.01 mg/dL, CI: −32.39, −9.64; *p* = 0.0003; *I*^2^ = 0) and total cholesterol (Figure 6; *N* = 3, *n* = 419; WMD = −14.40 mg/dL; CI: −26.51, −2.28; *p* = 0.02; *I*^2^ = 47) compared to placebo. No significant changes were noted for HDL cholesterol (Figure 3; *N* = 6, *n* = 565; *I*^2^ = 79) and LDL cholesterol (Figure 4; *N* = 3, *n* = 419; *I*^2^ = 89). None of the metformin studies examined VLDL cholesterol.

#### Off Label Lipid Lowering Agents: Others

For other off label lipid lowering agents, there was a statistically significant reduction in total cholesterol levels (Figure 6; *N* = 27, *n* = 1,322; WMD = −5.18 mg/dL, CI: −10.31, −0.05; *p* = 0.05; *I*^2^ = 41) along with a decreasing trend in levels of triglycerides that was nonsignificant (Figure 2; *N* = 28, n = 1,337; WMD = −11.06, CI: −23.08, 0.97; *p* = 0.07; *I*^2^ = 39). There were no significant differences for LDL cholesterol (Figure 4; *N* = 28, *n* = 1,301; *I*^2^ = 49), HDL cholesterol (Figure 3; *N* = 29, *n* = 1,351; *I*^2^ = 0), and VLDL cholesterol (Figure 5; *N* = 4; *n* = 223; *I*^2^ = 0).

### Secondary Outcomes: Additional Metabolic Measures

Cumulatively, the pharmacological interventions reviewed in this paper were associated with significant reductions in body weight (Supplementary Figure 1; *N* = 38, *n* = 2,380; WMD = −1.13 kg, CI: −2.18, −0.08; *p* = 0.03), BMI (Supplementary Figure 2; *N* = 36, *n* = 2,174; WMD = −0.42 kg/m^2^, CI: −0.85, 0.01; *p* = 0.05), and waist circumference (Supplementary Figure 3; *N* = 29, *n* = 1,532; WMD = −1.34 cm, CI: −2.34, −0.34; *p* = 0.009) compared to placebo. As for glucose-related parameters, interventions led to significant decreases in blood insulin (Supplementary Figure 4; *N* = 24, *n* = 1,636; WMD = −1.64 mIU/mL, CI: −2.76, −0.52; *p* = 0.004) and HOMA-IR (Supplementary Figure 5; *N* = 16, *n* = 867; WMD = −0.52, CI: −0.89, −0.15; *p* = 0.005) compared to placebo. Blood glucose levels showed a decreasing trend, but the difference was not significant (Supplementary Figure 6; *N* = 46, *n* = 3,048; WMD = −1.17 mg/dL, CI: −2.44, −0.11; *p* = 0.07). Differences in HbA1c levels were also not significant (Supplementary Figure 7; *N* = 19, *n* = 1,097). Total PANSS scores showed a trend toward improvement in the intervention group, but the difference again was not statistically significant (Supplementary Figure 8; *N* = 13, *n* = 1,005, WMD = −2.15; CI: −4.45, 0.16; *p* = 0.07). Finally, there were no significant differences in systolic blood pressure (Supplementary Figure 9; *N* = 16, *n* = 892) and diastolic blood pressure (Supplementary Figure 10; *N* = 15, *n* = 845).

#### Risk of Bias

Risk of bias in random sequence generation was deemed to be low in 29 studies, high in 1, and unclear in 18 (Supplementary Figure 11). Outcomes did not change significantly after the study with high risk of bias was removed.

## Discussion

In this systematic review and meta-analysis, we examined different pharmacological interventions used to treat antipsychotic-induced dyslipidemia in schizophrenia spectrum disorders. The 29 pharmacological interventions analyzed were cumulatively effective in lowering total cholesterol, LDL cholesterol, and triglycerides, while increasing HDL cholesterol. However, improvements were not significant with VLDL cholesterol. Amongst the subgroups analyzed, we found that antipsychotic switching/add-on proved most effective in improving lipid parameters commonly dysregulated in schizophrenia, namely triglycerides and HDL cholesterol (77). Notably, the off-label lipid lowering agent metformin was more promising than approved lipid lowering agents in decreasing triglycerides and total cholesterol levels. However, other off label agents only showed a trend in improving lipid parameters.

Our findings suggest that off label strategies can be effectively employed to ameliorate antipsychotic-induced dyslipidemia. In particular, metformin shows considerable promise, improving lipid parameters and showing consistent association with a decrease in triglycerides and total cholesterol levels. Similar findings for triglycerides and total cholesterol levels were previously demonstrated in a review by Jiang et al. (78), and in the context of schizophrenia would benefit through evidence specific to long-term outcomes. Prior studies indicate that a 40 mg/dL reduction in LDL cholesterol and triglycerides translates into a 20% and 4–5% decrease in risk for developing cardiovascular disease, respectively, independent of baseline risk (79). Given this, our review suggests that the available strategies for targeting dyslipidemia are inadequate, reinforcing the need for novel, more effective interventions. Furthermore, while our findings provide strong evidence for antipsychotic switch/add-on interventions, study duration ranged from 6 to 24 weeks, which does not provide adequate time to assess the long-term effects of these treatments on dyslipidemia. While aripiprazole and quetiapine are both second generation antipsychotics with less severe metabolic side-effects compared to others like olanzapine and clozapine, they have their own metabolic burden that cannot be ignored and needs to be better understood over the longer term (80, 81). Similarly, while our findings suggest that lipid lowering agents are not effective in improving dyslipidemia, these results may have been limited by the short duration of the included studies. The small number of studies also did not permit examination of the possible effects of dose of lipid-lowering agents.

Current studies provide general support for the potential effectiveness of pharmacological interventions, but further research is warranted to refining recommendations pertaining to individual treatments. Currently in many studies, lipid management was not a primary focus; of the 48 reviewed studies, only 26 identified lipid profile as a primary outcome measure, in contrast to 22 where it was positioned as a secondary outcome. More studies focused on this area of research sets the stage for additional insights and the increased power necessary to detect not only beneficial outcomes, but also the elucidation of specific variables contributing to effective treatment. At present, the significant heterogeneity among studies within intervention categories limit generalizations that can be made with respect to mechanisms or interacting variables. Factors affecting outcomes may include specific antipsychotic treatment, diagnosis and stage of illness, co-morbid health conditions, concomitant medications, and duration of antipsychotic and/or lipid intervention treatment. Differential pharmacological interventions may, in fact, vary as a function of patient population. Moreover, to date, many interventions are confined to a single study, precluding pooled data or comparisons between interventions. Our review restricted the population to schizophrenia patients, even though antipsychotics are used to treat patients with other psychiatric illnesses such as affective disorders who also share the metabolic burden (82) and may benefit from the reviewed interventions. Finally, while behavioral and lifestyle interventions remain first-line treatments for dyslipidemia, our review restricted the search to pharmacological interventions.

Given the prevalence of dyslipidemia in schizophrenia, along with the associated increased risk of metabolic complications and cardiovascular disease, it is imperative that such studies be undertaken. At present, dyslipidemia is often left untreated (13–15); indeed, the physical health of this population is generally overlooked while the focus is directed to managing psychotic symptoms (83, 84). The integration of psychiatric and medical care falls short at present (85); however, this overview of dyslipidemia, its prevalence and current treatment underscores the need to ensure a more comprehensive model of care be implemented.

## Data Availability Statement

The original contributions generated for the study are included in the article/Supplementary Material, further inquiries can be directed to the corresponding author/s.

## Author Contributions

PK, SMA, and MKH contributed to developing the original protocol. PK and KC-D contributed to the original screening, data extraction, risk of bias assessments, and writing the first draft of the manuscript (introduction, methods, and results). FP and JL wrote and registered the protocol with PROSPERO, re-ran the search, updated study selection and risk of bias, and contributed to final data extraction and synthesis prior to manuscript submission, as well as updating the first draft. LH assisted with editing and writing the final draft. SMA was involved in supervising all aspects of the review. GR and MKH contributed to editing the final draft. All authors contributed to the article and approved the submitted version.

## Conflict of Interest

MKH has received Alkermes consultation fees. GR has received advisory board support from HLS Therapeutics and consultant fees from Mitsubishi Tanabe Pharma Corporation. The remaining authors declare that the research was conducted in the absence of any commercial or financial relationships that could be construed as a potential conflict of interest.
