# Supplementary figures and images for: Pharmacological Interventions to Treat Antipsychotic-Induced Dyslipidemia in Schizophrenia Patients: A Systematic Review and Meta Analysis
Source: Front Psychiatry. 2021 Mar 17;12:642403. doi: 10.3389/fpsyt.2021.642403 (PMC8010007; doi:10.3389/fpsyt.2021.642403)

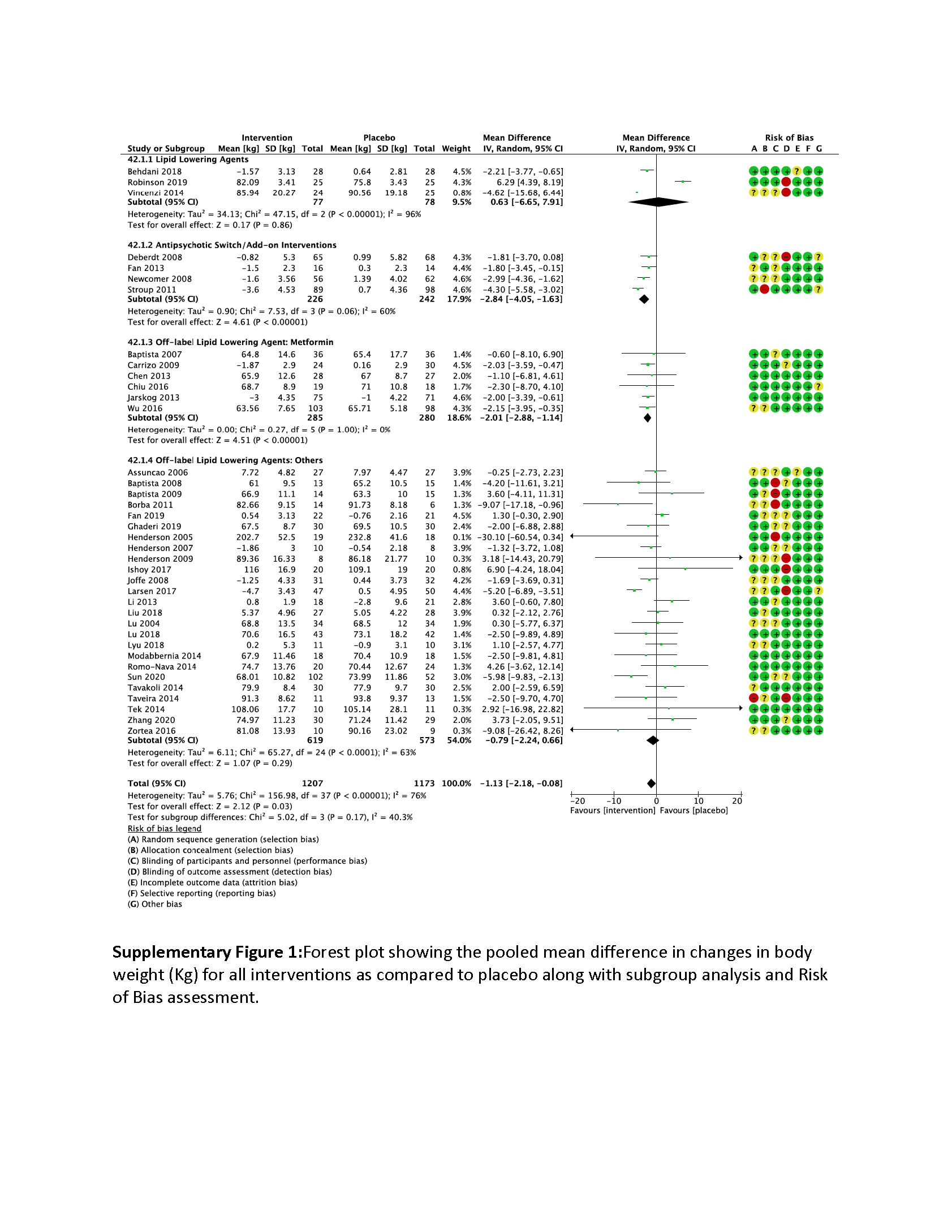

Supplement: Supplementary file 1 [file Image_1.TIFF]

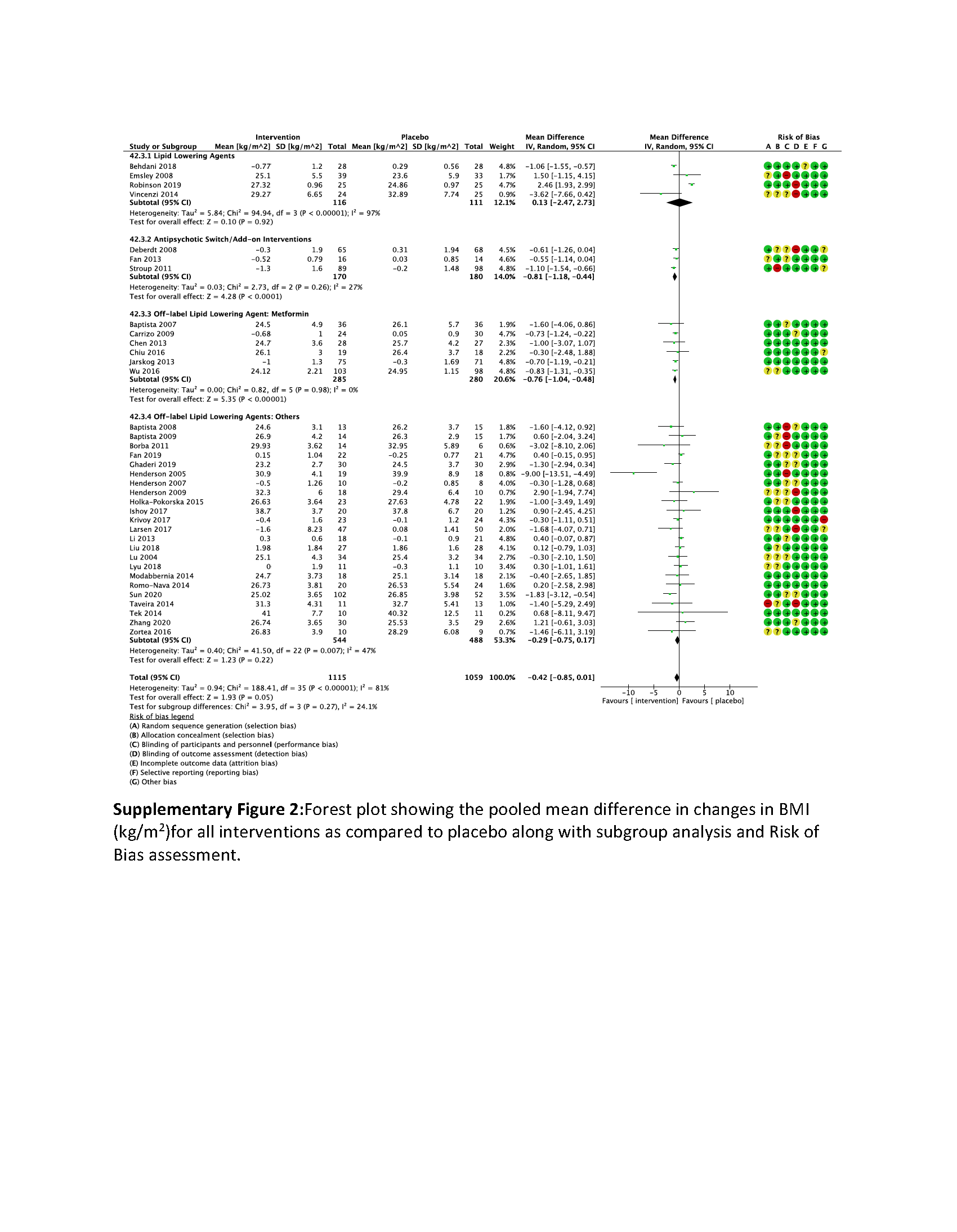

Supplement: Supplementary file 2 [file Image_2.TIFF]

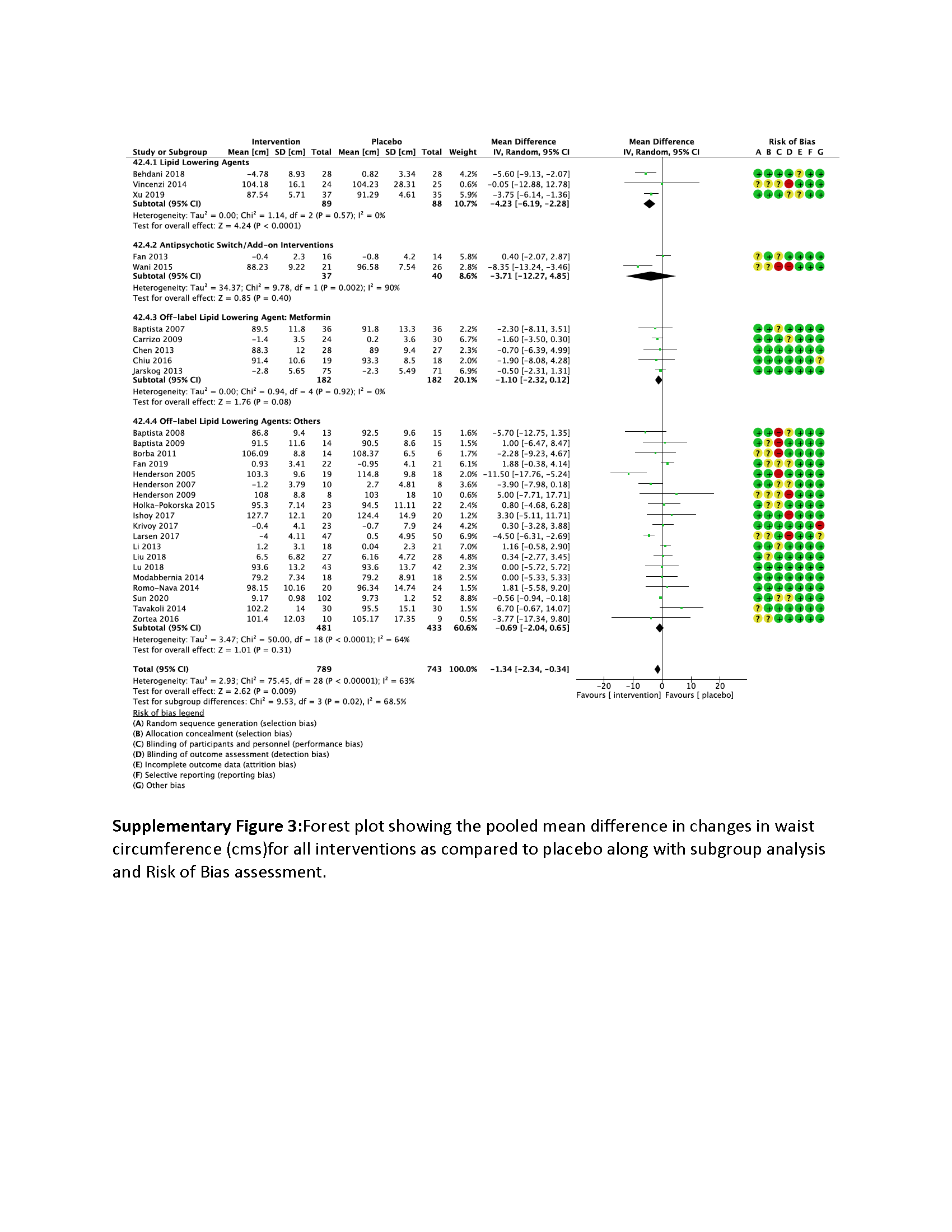

Supplement: Supplementary file 3 [file Image_3.TIFF]

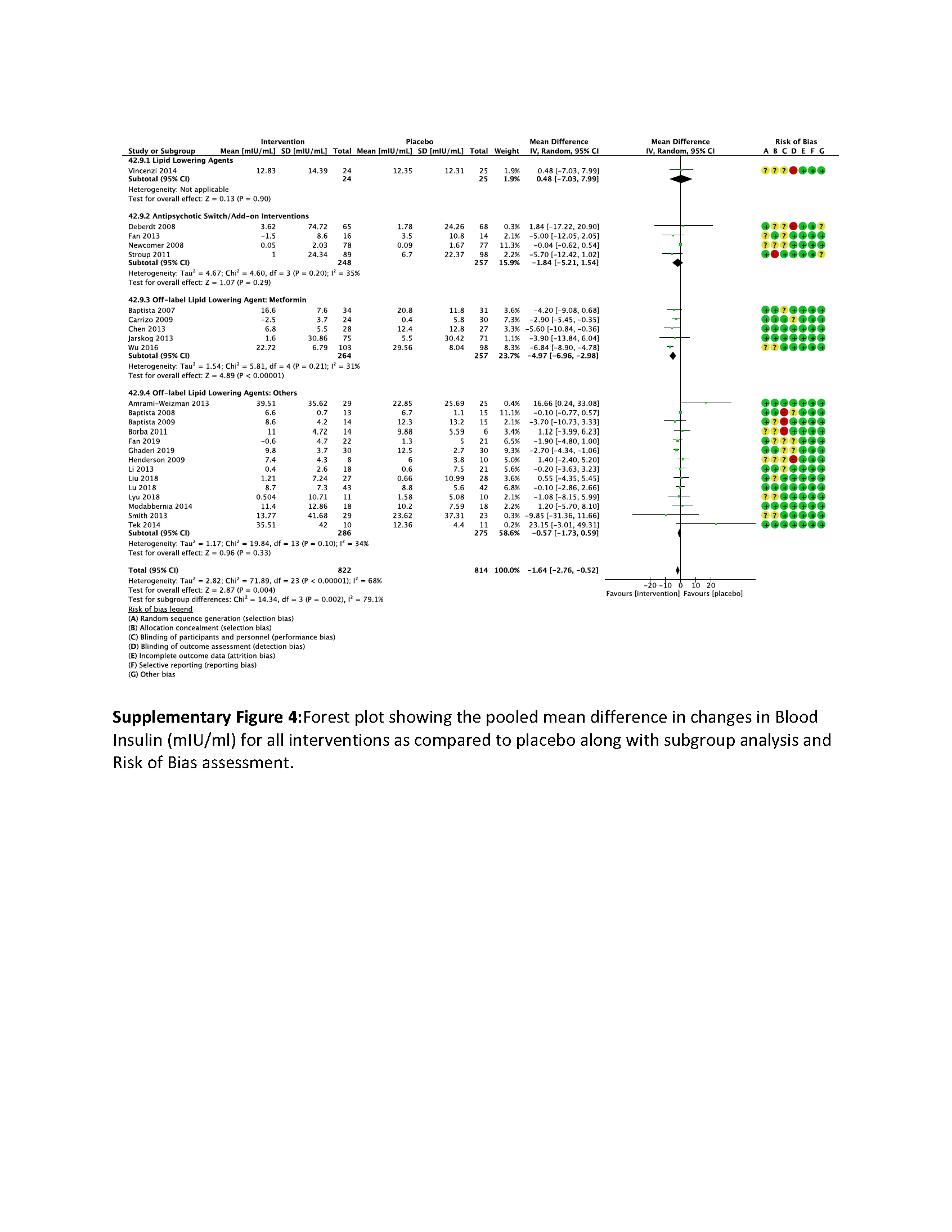

Supplement: Supplementary file 4 [file Image_4.TIFF]

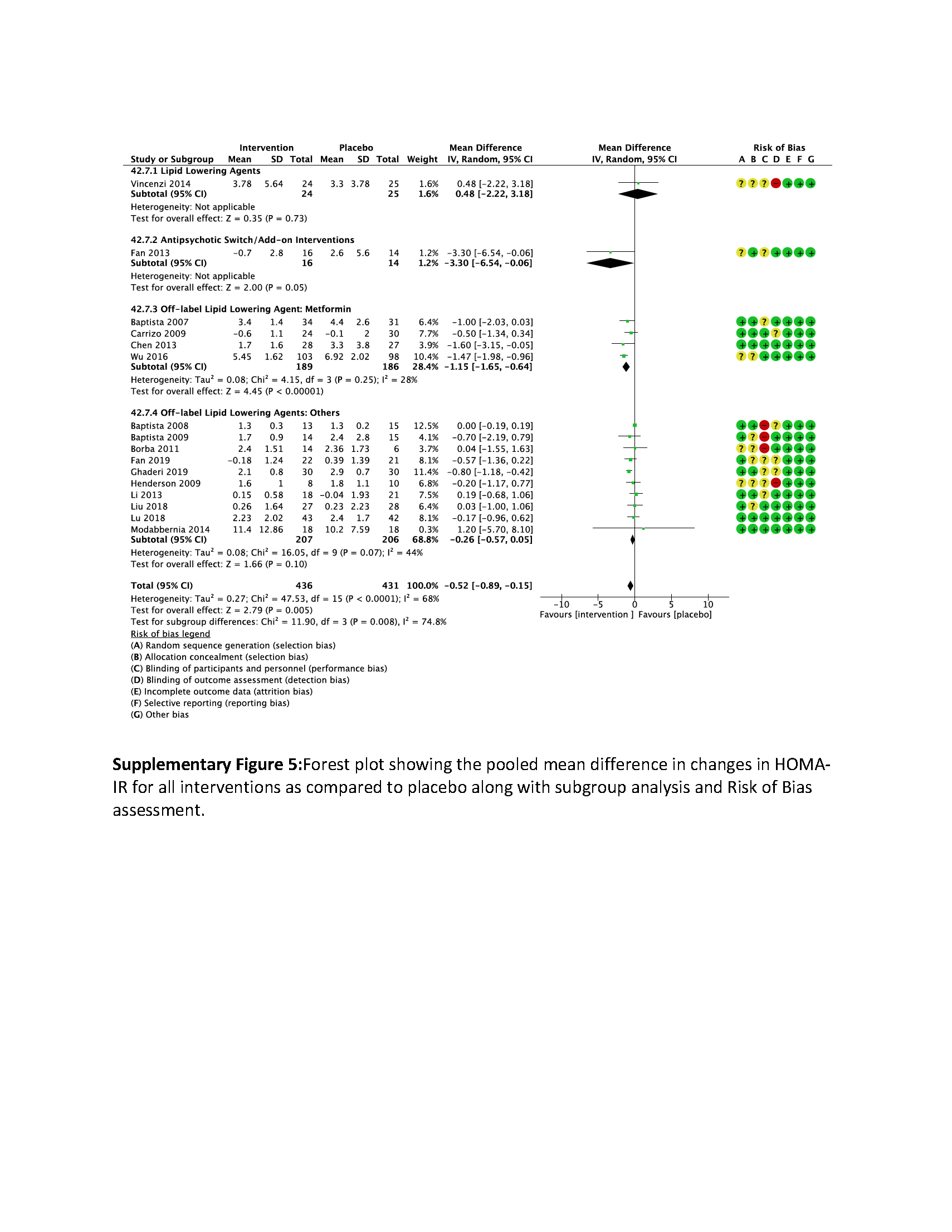

Supplement: Supplementary file 5 [file Image_5.TIFF]

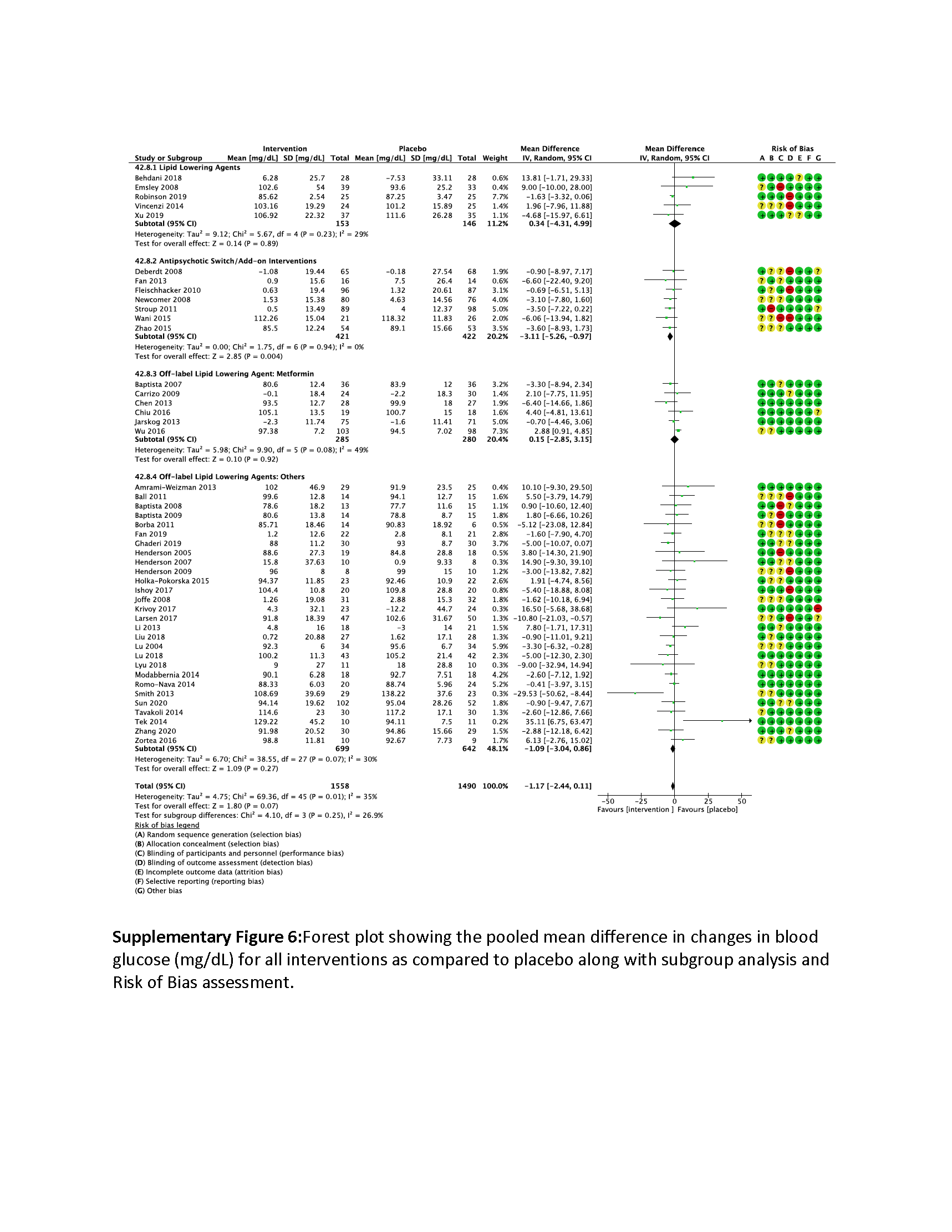

Supplement: Supplementary file 6 [file Image_6.TIFF]

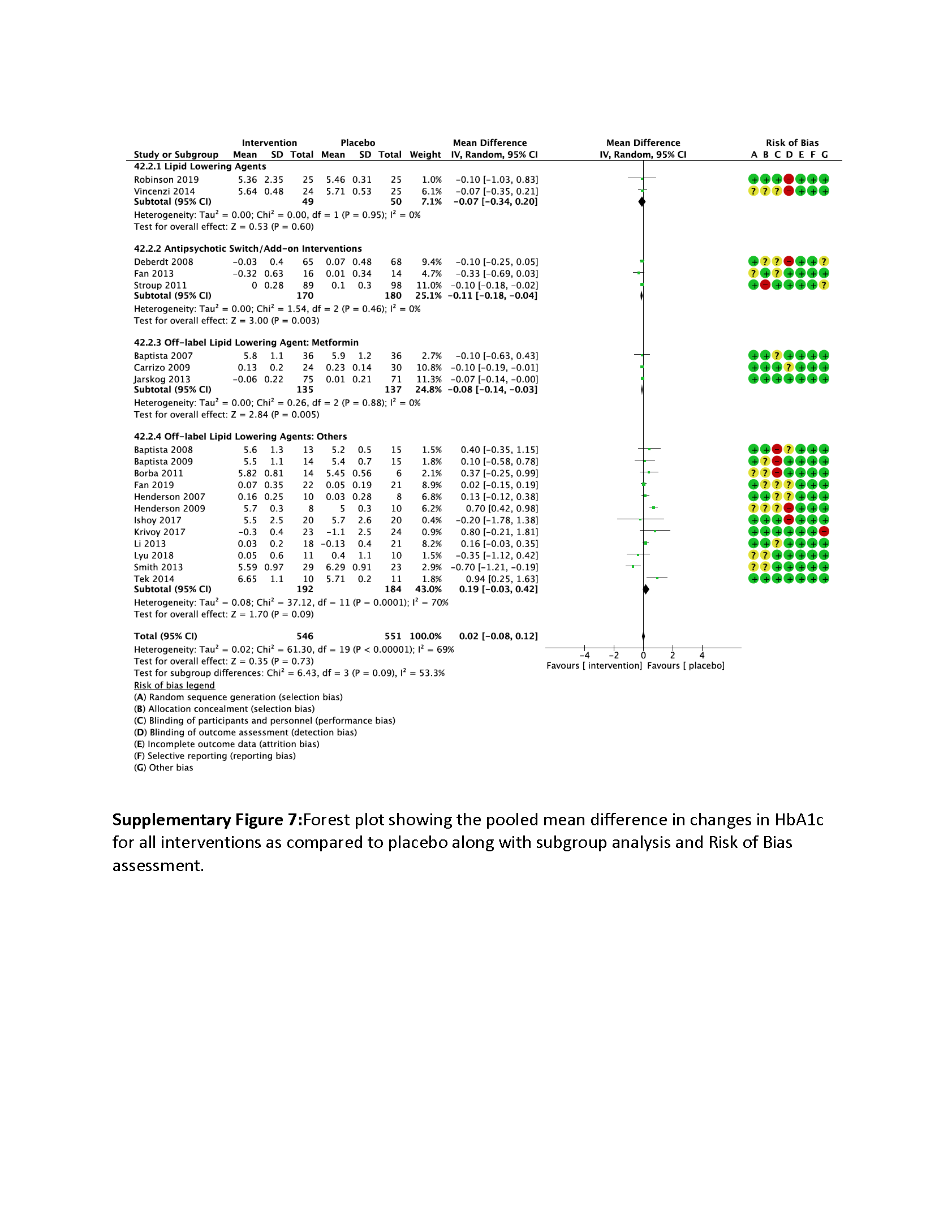

Supplement: Supplementary file 7 [file Image_7.TIFF]

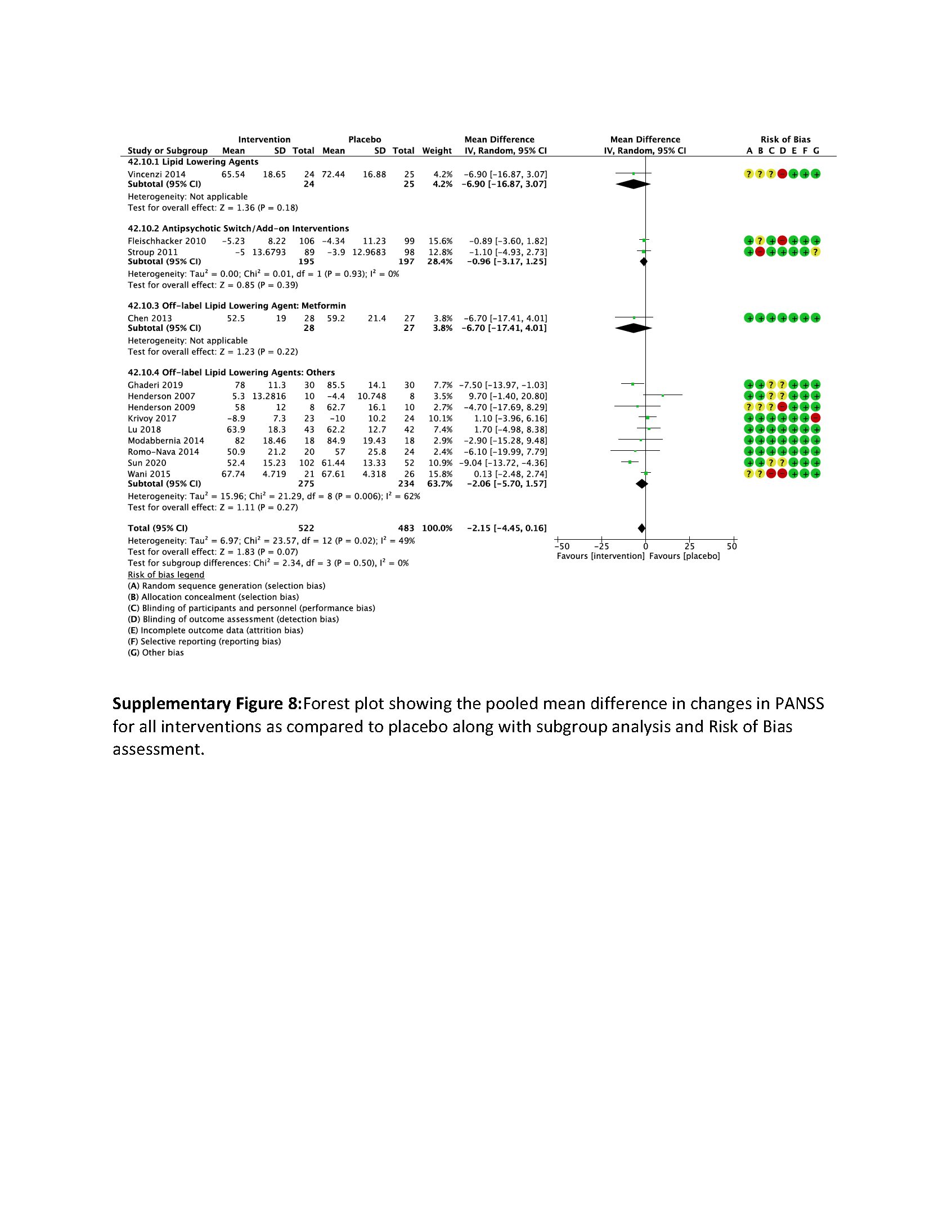

Supplement: Supplementary file 8 [file Image_8.TIFF]

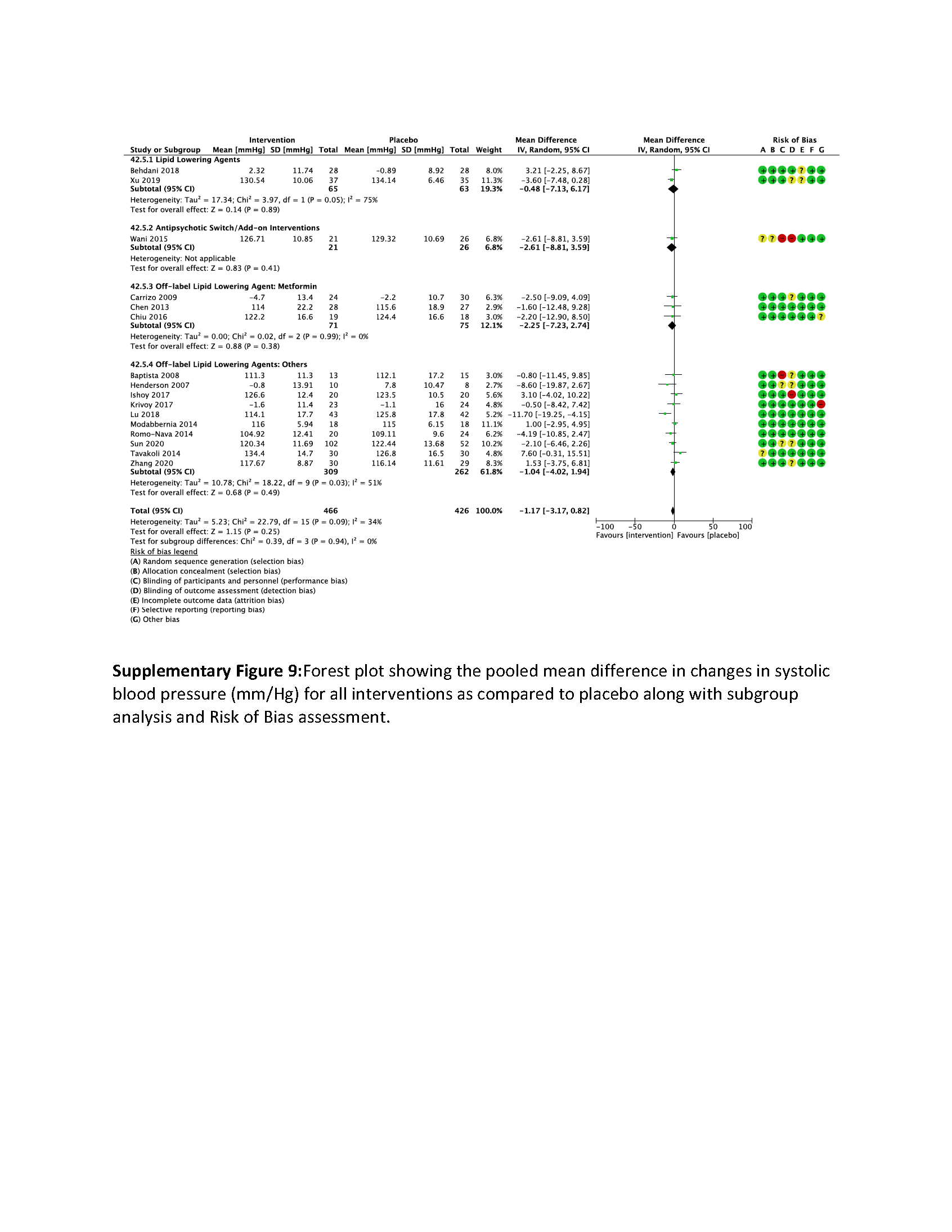

Supplement: Supplementary file 9 [file Image_9.TIFF]

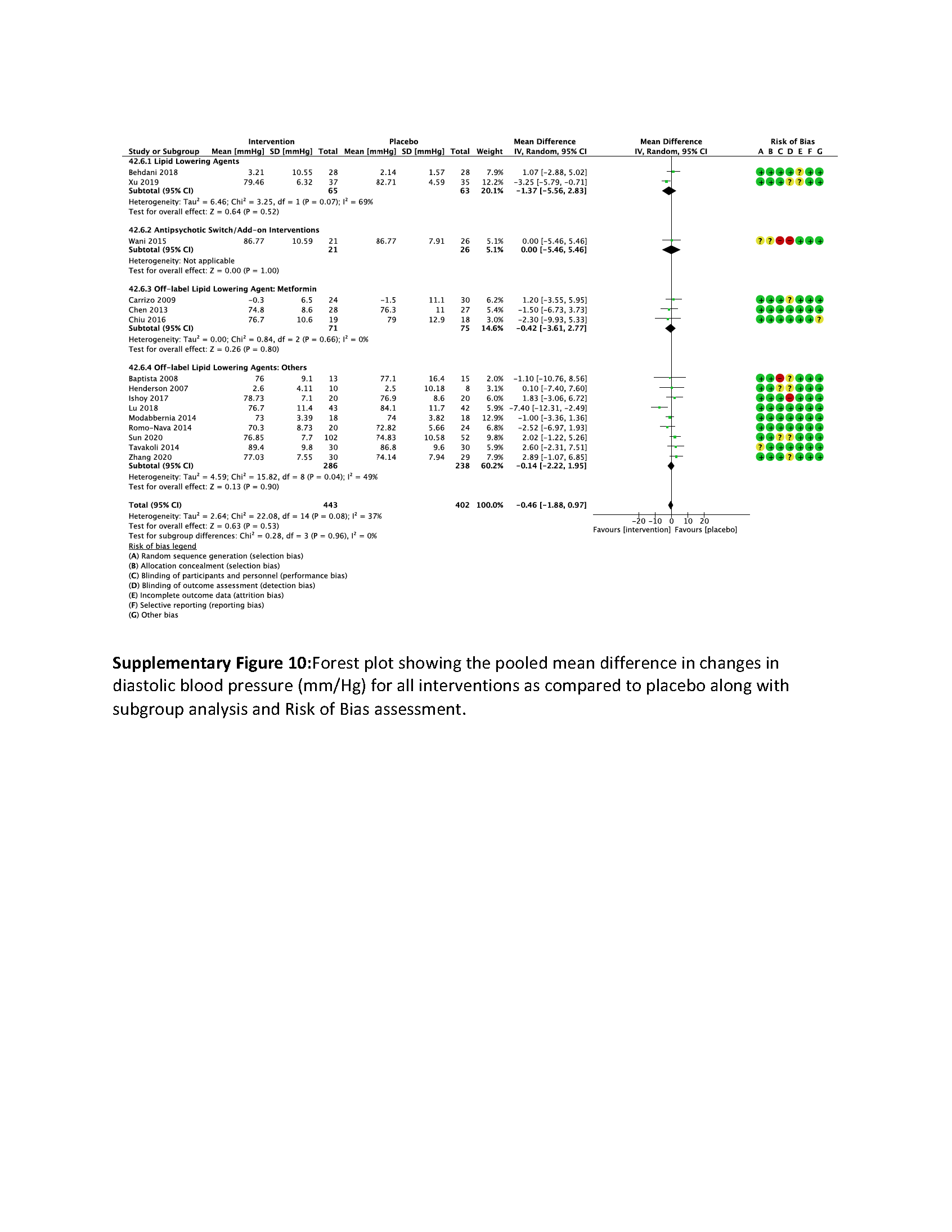

Supplement: Supplementary file 10 [file Image_10.TIFF]

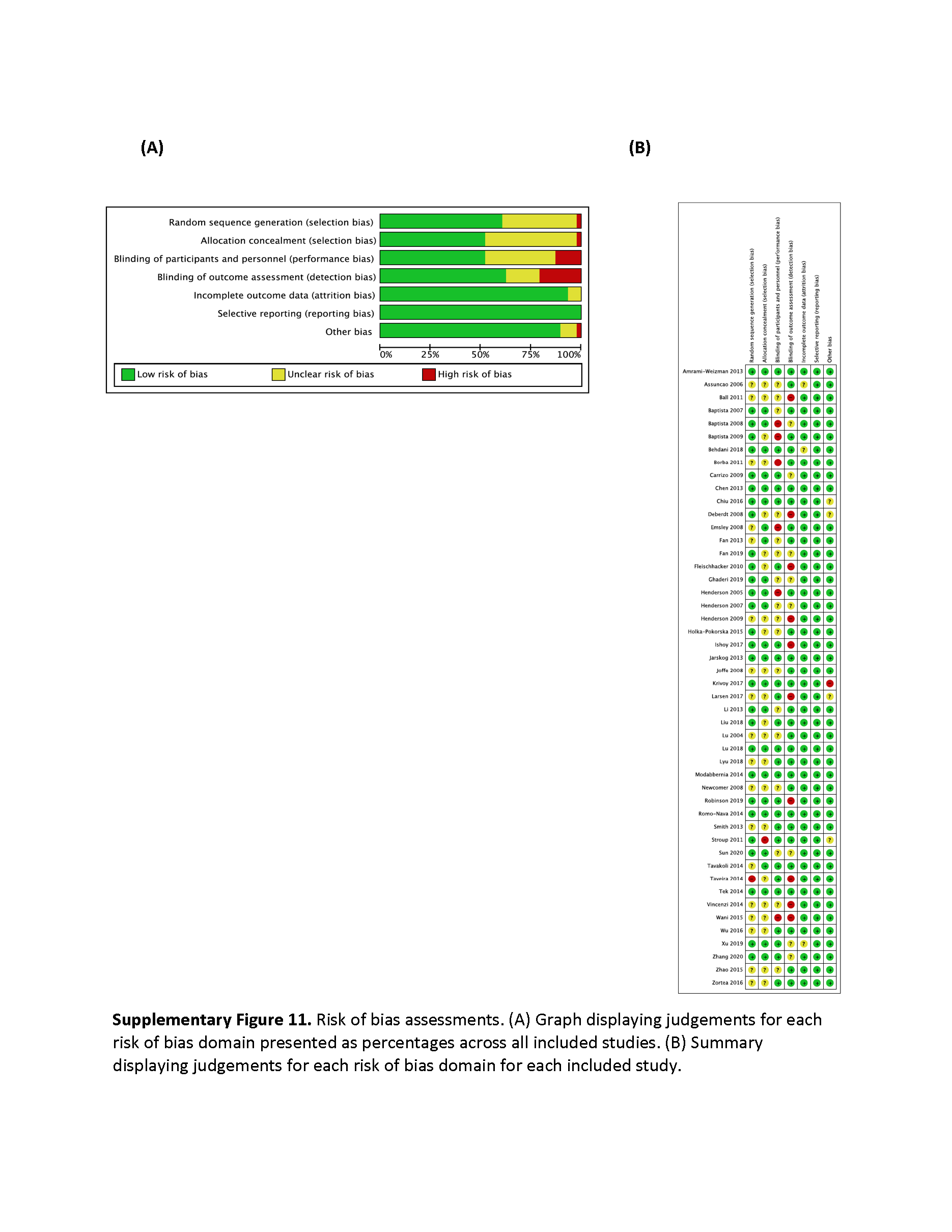

Supplement: Supplementary file 11 [file Image_11.TIFF]
