# Supplementary material for: Pharmacological Interventions to Treat Antipsychotic-Induced Dyslipidemia in Schizophrenia Patients: A Systematic Review and Meta Analysis
Source: Front Psychiatry. 2021 Mar 17;12:642403. doi: 10.3389/fpsyt.2021.642403 (PMC8010007; doi:10.3389/fpsyt.2021.642403)
Supplement: Supplementary file 12 [file Table_1.docx]

**Supplementary Table 1.** Summary of the studies included in the systematic review and meta-analysis. *Represents mean age for all participants, as age for the individual placebo and treatment groups were not available. LDL = low-density lipoprotein, HDL = high-density lipoprotein, VLDL = very low-density lipoprotein.

| Author Year | Blinding | Specific Diagnosis | Antipsychotic | Intervention | Dose (treatment group) | Study Duration (weeks) | Sex  Male (%) | Mean Age (SD) | | Main Outcomes |
| --- | --- | --- | --- | --- | --- | --- | --- | --- | --- | --- |
|  |  |  |  |  |  |  |  | **Treatment** | **Placebo** |  |
| *Lipid Lowering Agents* | | | | | | | | | | |
| Behdani et al., 2018 (1) | Double-blind | Schizophrenia | Clozapine | Omega-3 fatty acid (n = 28) Placebo (n = 28) | 4 g/day | 8 | 36 (64.3) | 37.2  (5.9) | 36.1  (7.9) | No significant differences in total, HDL, LDL cholesterol and triglycerides between the treatment and placebo groups |
| Emsley et al., 2008 (2) | Double-blind | Schizophrenia, Schizoaffective disorder | Haloperidol, Fluphenazine, Flupenthixol, Clopenthixol | Omega 3 fatty acid, EPA (eicosapentaenoic acid) 2g/day (n = 39) Placebo (n = 33) | 2 g/day | 12 | 50  (69.4) | 42  (10) | 43  (10) | No significant differences in total, HDL, LDL cholesterol and triglycerides between the treatment and placebo groups |
| Robinson et al., 2019 (3) | Open-label | Schizophrenia, Schizophreniform, Schizoaffective disorder, Psychosis not others specified, Bipolar 1 disorder | Risperidone | Omega 3 (n = 25) Placebo (n = 25) | 3-6 mg/day | 16 | 35  (70) | 22.0  (5.3) | 21.1  (5.3) | Significantly greater increase in total cholesterol in placebo group. HDL cholesterol non-significantly increased in placebo group and decreased in treatment group, with trend-level group differences (p = 0.0738). No significant differences for LDL cholesterol and triglycerides. |
| Vincenzi et al., 2014 (4) | Double-blind | Schizophrenia, Schizoaffective | Clozapine, Olanzapine, Aripiprazole, Quetiapine, Ziprasidone, Paliperidone, Risperidone | Pravastatin (n = 24) Placebo (n = 25) | 40 mg/day | 12 | 38 (63.3) | 42.6  (11.0) | 44.5 (12.6) | Significantly greater decrease in LDL cholesterol and total cholesterol in the treatment group compared to placebo. No significant differences for triglycerides and HDL cholesterol. |
| Xu et al., 2019 (5) | - | Schizophrenia | Olanzapine | Omega-3 fatty acid (n = 37) Placebo (n = 35) | 720 mg eicosapent-aenoic acid + 480 mg docosahex-aenoic acid /day | 12 | 50 (62.5) | 28.0  (3.1) | 28.8  (4.7) | Significant reduction in triglyceride levels in treatment group (group x time effect). |
| *Antipsychotic Switch/Add-on Interventions* | | | | | | | | | | |
| Deberdt et al., 2008 (6) | Double-blind | Schizophrenia, Schizoaffective disorder | olanzapine, quetiapine | Switch to quetiapine (n = 65) Placebo (n = 68) | Mean modal dose: 439.7 mg/day | 24 | - | 42.5  (11.5) | 45.4  (9.4) | No significant differences for triglycerides, HDL, LDL, and total cholesterol. |
| Fan et al., 2013 (7) | Double-blind | Schizophrenia, Schizoaffective disorder | Clozapine | Aripiprazole add-on (n = 16) Placebo (n = 14) | 15 mg/day | 6 | 22 (73.3) | 44.3  (8.2) | 44.2  (8.9) | Significant reduction in LDL cholesterol in treatment group, compared to placebo group. No significant group differences for total cholesterol, HDL, and triglycerides. |
| Fleischhacker et al., 2010 (8) | Double-blind | Schizophrenia | Clozapine, aripiprazole | Aripiprazole add-on (n = 97) Placebo (n =88) | 5-15 mg/day | 16 | 134 (64.7) | 37.6  (10.9) | 40.5  (9.9) | Significant decrease in total and LDL cholesterol in treatment group, compared to placebo. No group differences for HDL cholesterol and triglycerides. |
| Newcomer et al., 2008 (9) | Double-blind | Schizophrenia, Schizoaffective disorder | Olanzapine, aripiprazole | Switch to aripiprazole (n = 80) Placebo (n = 76) | 10-30 mg/day | 16 | 111 (64.2) | 39.7  (10.1) | 38.7  (10.1) | Significant increases in HDL cholesterol and decreases in triglycerides, non-HDL and total cholesterol with treatment, compared to placebo. No group differences for LDL cholesterol. |
| Stroup et al., 2011 (10) | - | Schizophrenia, Schizoaffective disorder | Olanzapine, quetiapine, risperidone, aripiprazole | Switch to aripiprazole (n = 89) Placebo (n = 98) | 5-30 mg/day | 24 | 137 (63.7) | 40.0  (11.7) | 42.0  (10.5) | Significant decrease in triglycerides and total cholesterol in switch compared to placebo group. No group differences for changes in HDL or LDL cholesterol. |
| Wani et al., 2015 (11) | Double-blind | Schizophrenia | Olanzapine | Switch to aripiprazole (n = 21) Placebo (n = 26) | 10-30 mg/day | 24 | 39  (62.9) | 29.8  (5.2) | 29.7  (5.0) | HDL significantly increased in treatment group and significantly decreased in placebo group. Triglycerides significantly increased in placebo group. No significant change in triglycerides in treatment group. |
| Zhao et al., 2015 (12) | Open-label | Schizophrenia, Schizoaffective disorder | Risperidone | Aripiprazole add-on (n = 54) Placebo (n = 53) | 10 mg/day | 8 | 46  (40.7) | 28.9  (7.8) | 30.4  (8.3) | Significant treatment x time effect on LDL cholesterol. No significant differences in changes in total and HDL cholesterol, and triglycerides. |
| *Off Label Lipid Lowering Agent: Metformin* | | | | | | | | | | |
| Baptista et al., 2007 (13) | Double-blind | Schizophrenia, Bipolar disorder | Olanzapine | Metformin (n = 36) Placebo (n = 36) | 850-2550 mg/day | 12 | 42  (58.3) | 42.4  (11.7) | 43.2  (14.5) | HDL cholesterol significantly decreased in treatment group, without significant between-group differences. LDL cholesterol tended to increase in treatment group (between-group difference, p = 0.055). No significant differences for total cholesterol and triglycerides. |
| Carrizo et al., 2009 (14) | Double-blind | Schizophrenia, Bipolar I disorder, Schizophreniform disorder | Clozapine | Metformin (n = 24) Placebo (n = 30) | 500-1000 mg daily | 14 | - | 39.6  (9.7) | 38.3  (8.7) | HDL cholesterol significantly increased in treatment group and decreased in placebo group, with significant between-group differences. No significant differences for non-HDL cholesterol. |
| Chen et al., 2013 (15) | Double-blind | Schizophrenia, Schizoaffective disorder | Clozapine | Metformin (n = 28) Placebo (n = 27) | 1500 mg/day | 24 | 28 (50.9) | 41.8  (7.2) | 41.4  (10.2) | No significant group differences. Significant reduction in HDL cholesterol in both treatment and placebo groups. No significant differences for triglycerides. |
| Chiu et al., 2016 (16) | Double-blind | Schizophrenia, Schizoaffective disorder | Clozapine | Metformin (n = 19) Placebo (n = 18) | 1000 mg/day | 12 | 24  (43.6) | 50.3  (6.1) | 44.2  (10.0) | No significant differences in HDL cholesterol and triglycerides between the treatment and placebo groups |
| Jarskog et al., 2013 (17) | Double-blind | Schizophrenia, Schizoaffective disorder | Various | Metformin (n = 75) Placebo (n = 71) | 1000 – 2000 mg/day | 16 | 141  (69.2) | 41.4  (11.5) | 45.0  (10.3) | Triglycerides decreased with treatment and increased with placebo, with significant group differences. Total cholesterol reduced with treatment, with trend level group differences (p = 0.057). No differences for HDL and LDL cholesterol. |
| Wu et al., 2016 (18) | Double-blind | Schizophrenia | Olanzapine, Clozapine, Sulpiride, Risperidone | Metformin (n = 103) Placebo (n = 98) | 1000 mg/day | 24 | 81  (40.2) | 26.1  (4.7) | 25.5  (4.4) | Significant decrease in total cholesterol, LDL, and triglycerides and increase in HDL cholesterol in treatment group compared to placebo. |
| *Off Label Lipid Lowering Agents: Others* | | | | | | | | | | |
| Amrami-Weizman et al., 2013 (19) | Double-blind | Schizophrenia, Schizophreniform disorder | Olanzapine | Reboxetine (n = 29) Placebo (n = 25) | 4 mg/day, b.i.d. | 6 | 30  (55.6) | 30.0  (8.3) | 29.2  (7.0) | Triglycerides significantly decreased in treatment group and increased in placebo group, with group differences. No between-group differences for total, LDL and HDL cholesterol. |
| Assuncao et al., 2006 (20) | Double-blind | Schizophrenia, Schizoaffective disorder, Schizophreniform disorder | Olanzapine | Nizatidine (n = 27) Placebo (n = 27) | 300 mg b.i.d. | 12 | 32  (59.3) | 35.5  (12.4) | 34.9  (12.2) | No significant group differences in changes in triglycerides and total, HDL, and LDL cholesterol |
| Ball et al., 2011 (21) | Double-blind | Schizophrenia, Schizoaffective disorder | Olanzapine or Clozapine | Atomoxetine (n =14) Placebo (n = 15) | 80-120 mg/day | 24 | 25  (69.4) | 47.8  (8.4) | 46.1  (9.4) | Decreasing trend for lower total cholesterol (p = 0.06) for both groups. Increasing trend for HDL (p = 0.08) in the treatment group, compared to placebo (p = 0.08). No significant differences for LDL cholesterol and triglycerides. |
| Baptista et al., 2008 (22) | Double-blind | Schizophrenia | Olanzapine | Metformin + sibutramine (n = 13) Placebo (n = 15) | Metformin 1700 mg  Sibutramine 20 mg/day | 12 | 14  (50.0) | 45.6  (7.8) | 49.4 (12.0) | No significant group differences in changes in triglycerides and total, HDL, and LDL cholesterol |
| Baptista et al., 2009 (23) | Double-blind | Schizophrenia | Olanzapine | Rosiglitazone (n = 14) Placebo (n= 15) | 4-8 mg/day | 12 | 14  (48.3) | 44.2  (9.1) | 51.4  (6.8) | No significant differences in total, HDL, LDL cholesterol and triglycerides between the treatment and placebo groups |
| Borba et al., 2011 (24) | Double-blind | Schizophrenia, Schizoaffective disorder | Olanzapine, Clozapine, Quetiapine, Risperidone | Ramelteon (n = 14) Placebo (n = 6) | 8 mg/day | 8 | 13  (65) | 49  (7) | 56  (9) | Total cholesterol decreased significantly in treatment group and nonsignificantly in placebo group. LDL cholesterol decreased nonsignificantly in the treatment group (p = 0.1) |
| Fan et al., 2019 (25) | Double-blind | Schizophrenia, Schizoaffective disorder | Olanzapine, clozapine | Telmisartan (n = 22) Placebo (n = 21) | 5–30 mg/day | 12 | 34  (79.1) | 41.5  (12.3) | 44.4  (11.5) | No significant differences in total, HDL, LDL cholesterol and triglycerides between the treatment and placebo groups |
| Ghaderi et al., 2019 (26) | Double-blind | Schizophrenia | Chlorpromazine | Vitamin D + Probiotic (n = 30) Placebo (n = 30) | 50,000 IU vitamin D3 every 2 weeks + 8 × 109 CFU/day probiotic | 12 | 56  (93.3) | 44.8  (8.3) | 43.2  (6.0) | Treatment significantly reduced triglycerides, VLDL, total, LDL cholesterol, compared to placebo. No significant differences for HDL cholesterol. |
| Henderson et al., 2005 (27) | Double-blind | Schizophrenia, Schizoaffective disorder | Olanzapine | Sibutramine (n = 19) Placebo (n = 18) | 5-15 mg/day | 12 | 23  (62.2.) | 43.2  (10.6) | 40.7  (9.9) | No significant group differences in changes in triglycerides, total, HDL, and LDL cholesterol. |
| Henderson et al., 2007 (28) | Double-blind | Schizophrenia, Schizoaffective disorder | Clozapine | Sibutramine (n = 10) Placebo (n = 8) | 5-15 mg/day | 12 | 16  (76.2) | 41  (10) | 39  (10) | No significant group differences in changes in triglycerides and total, HDL, and LDL cholesterol |
| Henderson et al., 2009 (29) | Double-blind | Schizophrenia, Schizoaffective disorder | Clozapine | Rosiglitazone (n = 8) Placebo (n = 10) | 4 mg/day | 8 | 13 (72.2) | 39.2 (9.2) | 39.7 (7.4) | No significant within- or between-group differences in triglycerides and total, HDL, and LDL cholesterol within both groups. |
| Holka-Pokorska et al., 2015 (30) | Double-blind | Schizophrenia, Schizoaffective disorder | Olanzapine | DHEA (dehydroepiandrosterone) (n = 23) Placebo (n = 22) | 100 mg/day | 12 | 55 (100) | - | - | No significant differences in total, HDL, LDL cholesterol and triglycerides between the treatment and placebo groups |
| Ishoy et al., 2017 (31) | Double-blind | Schizophrenia, Schizoaffective disorder | Perphenazine, zuclopenthixol, chlorprothixene, clozapine, olanzapine, aripiprazole, risperidone, paliperidone, quetiapine, ziprasidone, amisulpride sertindole | Subcutaneous exenatide (n = 20) Placebo (n = 20) | 2 mg/week | 12 | 20 (50.0) | 37.4  (10.7) | 34.4 (10.6) | Significant increases in total, LDL, VLDL, and HDL cholesterol, and triglycerides for both groups, without significant group differences |
| Joffe et al., 2008 (32) | Double-blind | Any "serious mental condition" treated by clozapine or olanzapine | Clozapine or olanzapine | Orlistat (n = 31) Placebo (n = 32) | 120 mg t.i.d. | 16 | 41 (65.1) | 38.3  (9.4) | 37.1  (9.7) | LDL decreased significantly with placebo, with a similar trend in treatment (p = 0.051) but no group differences. No differences for total and HDL cholesterol. |
| Krivoy et al., 2017 (33) | Double-blind | Schizophrenia | Clozapine | Vitamin D (n = 23) Placebo (n = 24) | 14,000 IU | 8 | 32  (68) | 39.4  (9.6) | 42.5 (11.2) | No significant group differences for changes in HDL cholesterol and triglycerides. |
| Larsen et al., 2017 (34) | Double-blind | Schizophrenia spectrum disorder | Clozapine or olanzapine | Liraglutide (n = 47) Placebo (n = 50) | 1.2-1.8 mg/day | 16 | 60 (61.9) | 42.1  (10.7) | 43  (10.5) | Significant decrease in total and LDL cholesterol in treatment group compared to placebo. No differences for HDL and VLDL cholesterol. |
| Li et al., 2013 (35) | Double-blind | Schizophrenia, Schizoaffective disorder | Olanzapine, "others" | Intranasal Insulin (n = 18) Placebo (n = 21) | 40 IU q.i.d. | 8 | 32 (82) | 48.7  (9.7) | 43.3  (9.6) | No significant differences in total, HDL, LDL cholesterol and triglycerides between the treatment and placebo groups |
| Liu et al., 2018 (36) | Double-blind | Schizophrenia | Risperidone | Minocycline (n = 27) Placebo (n = 28) | 200 mg/day | 16 | 32 (58.2) | 26.7  (5.5) | 28.9  (7.0) | No significant differences in total, HDL, LDL cholesterol and triglycerides between the treatment and placebo groups |
| Lu et al, 2004 (37) | Open-label | Schizophrenia | Clozapine | Fluvoxamine (n = 34) Placebo (n = 34) | 30-45 mg/day | 12 | 20 (29.4) | 32.9  (8.5) | 35.1  (9.4) | Triglycerides significantly increased in placebo but not treatment group. No significant changes in total cholesterol. |
| Lu et al., 2018 (38) | Double-blind | Schizophrenia | Clozapine | Fluvoxamine (n = 43) Placebo (n = 42) | 50 mg/day | 12 | 61 (71.8) | 43.6  (8.1) | 46.2  (9.8) | TG significantly increased in the placebo group, without significant between-group differences. No significant differences for total, HDL, and LDL cholesterol. |
| Lyu et al., 2018 (39) | Double-blind | Schizophrenia | Various | Naltrexone and bupropion (n = 11) Placebo (n = 10) | naltrexone 25 mg/day + bupropion (300 mg/day) | 24 | 21 (100) | 54.3  (5.7) | 56.2  (4.8) | No significant group differences in changes in triglycerides, HDL, LDL cholesterol. |
| Modabbernia et al., 2014 (40) | Double-blind | Schizophrenia | Olanzapine | Melatonin (n = 18) Placebo (n = 18) | 3 mg/day | 8 | 25 (69.4) | 32.7  (7.3) | 32.8  (8.2) | Significant group difference in changes in triglycerides. No significant group differences in changes in total, HDL, and LDL cholesterol. |
| Romo-Nava et al., 2014 (41) | Double-blind | Schizophrenia, Bipolar Type I | Quetiapine, Olanzapine, Risperidone, Clozapine | Melatonin (n = 20) Placebo (n = 24) | 5 mg/day | 8 | 22 (50.0) | 30.6  (7.6) | 28.6  (9) | No significant effect of treatment on triglycerides, HDL, LDL, and total cholesterol. |
| Smith et al., 2013 (42) | Double-blind | Schizophrenia, Schizoaffective disorder | Olanzapine, Clozapine, other 1^st^ and 2^nd^ generation antipsychotics | Pioglitazone (n = 29) Placebo (n = 23) | 30-45 mg/day | 12 | 48 (88.9) | 47.17  (9.1) | 48.46  (10.7) | Significant decreases in triglycerides and increases in HDL cholesterol in treatment group, with opposite trend in placebo group. No significant effects on LDL, total cholesterol, and triglycerides. |
| Sun et al., 2020 (43) | Blind (level not specified) | Schizophrenia | Clozapine, olanzapine, risperidone, quetiapine, or combination of two drugs | Liuyu decoction (traditional Chinese medicine) (n = 102) Placebo (n = 52) | 100 ml twice/day** | 12 | 87 (56.5) | * 45  (16) | *45  (16) | No significant differences for total, HDL, and LDL cholesterol. |
| Tavakoli et al., 2014 (44) | Double-blind | Schizophrenia | 1st generation, 2nd generation antipsychotics, or mixed | Celery, Dill and Green Tea (n = 30) Placebo (n = 30) | Celery seed extract powder 20 mg; dill seed extract 40 mg; green tea extract 150 mg; t.i.d. | 12 | 0  (0.0) | 47.3  (8.6) | 46.4  (9.6) | Significantly greater increase in HDL cholesterol with treatment, compared to placebo. No significant differences for LDL cholesterol and TG. |
| Taveira et al., 2014 (45) | Double-blind | Schizophrenia, Schizoaffective disorder | Olanzapine | Naltrexone (n = 11) Placebo (n = 13) | 50 mg/day | 12 | 20  (66.7) | 43.6  (11.2) | 45.6 (10.8) | HDL cholesterol and triglycerides significantly decreased in both groups, without significant between-group differences. Decreasing trend in total cholesterol for both groups (p = 0.08). No significant differences for LDL cholesterol. |
| Tek et al., 2014 (46) | Double-blind | Schizophrenia, Schizoaffective disorder | Various | Naltrexone (n = 10) Placebo (n = 11) | 25mg/day | 8 | 0  (0.0) | 50.0  (9.6) | 41.4  (11.6) | No significant within- or between-group differences in triglycerides and total, HDL, and LDL cholesterol. |
| Zhang et al., 2020 (47) | Double-blind | Schizophrenia | Clozapine, olanzapine, risperidone, quetiapine, sulpiride | Konjac powder (n=30) Placebo (n = 29) | 2 g of konjac flour t.i.d. | 4 | 33  (55.9) | 31.5  (10.3) | 32.6  (9.5) | Total cholesterol non-significantly decreased in treatment group and significantly increased in placebo group, with significant group differences. No significant differences for triglycerides, HDL and LDL cholesterol. |
| Zortea et al., 2016 (48) | Double-blind | Schizophrenia | Clozapine | Resveratrol (n = 10) Placebo (n = 9) | 200 mg/day | 4 | 19  (100) | 46.4  (11.2) | 41  (7.9) | No significant group differences. Significant increase in total cholesterol, LDL cholesterol, and triglycerides as well as reduction in HDL cholesterol in placebo group. Triglycerides significantly decreased in the treatment group. |

** For 500 mL of Liuyu Decoction, there are 12 g of Pinellia ternate, Gardenia, Cyperus, Fructus Aurantii, Atractylodes macrocephala, Magnolia officinalis, and tangerine peel; 15 g of Sichuan dome, Poria cocos, and Amomum kernel; and 6 g of licorice 6 g.

1. F. Behdani, S. N. Roudbaraki, M. Saberi-Karimian, M. Tayefi, P. Hebrani, A. Akhavanrezayat, S. V. Amlashi, G. A. Ferns and M. Ghayour-Mobarhan: Assessment of the efficacy of omega-3 fatty acids on metabolic and inflammatory parameters in patients with schizophrenia taking clozapine and sodium valproate. *Psychiatry Res*, 261, 243-247 (2018) doi:10.1016/j.psychres.2017.12.028

2. R. Emsley, D. J. Niehaus, P. P. Oosthuizen, L. Koen, B. Ascott-Evans, B. Chiliza, S. J. van Rensburg and R. M. Smit: Safety of the omega-3 fatty acid, eicosapentaenoic acid (EPA) in psychiatric patients: results from a randomized, placebo-controlled trial. *Psychiatry Res*, 161(3), 284-91 (2008) doi:10.1016/j.psychres.2007.06.029

3. D. G. Robinson, J. A. Gallego, M. John, L. A. Hanna, J. P. Zhang, M. L. Birnbaum, J. Greenberg, M. Naraine, B. D. Peters, R. K. McNamara, A. K. Malhotra and P. R. Szeszko: A potential role for adjunctive omega-3 polyunsaturated fatty acids for depression and anxiety symptoms in recent onset psychosis: Results from a 16 week randomized placebo-controlled trial for participants concurrently treated with risperidone. *Schizophr Res*, 204, 295-303 (2019) doi:10.1016/j.schres.2018.09.006

4. B. Vincenzi, S. Stock, C. P. Borba, S. M. Cleary, C. E. Oppenheim, L. J. Petruzzi, X. Fan, P. M. Copeland, O. Freudenreich, C. Cather and D. C. Henderson: A randomized placebo-controlled pilot study of pravastatin as an adjunctive therapy in schizophrenia patients: effect on inflammation, psychopathology, cognition and lipid metabolism. *Schizophr Res*, 159(2-3), 395-403 (2014) doi:10.1016/j.schres.2014.08.021

5. F. Xu, W. Fan, W. Wang, W. Tang, F. Yang, Y. Zhang, J. Cai, L. Song and C. Zhang: Effects of omega-3 fatty acids on metabolic syndrome in patients with schizophrenia: a 12-week randomized placebo-controlled trial. *Psychopharmacology*, 236(4), 1273-1279 (2019) doi:10.1007/s00213-018-5136-9

6. W. Deberdt, I. Lipkovich, A. N. Heinloth, L. Liu, S. Kollack-Walker, S. E. Edwards, V. P. Hoffmann and T. A. Hardy: Double-blind, randomized trial comparing efficacy and safety of continuing olanzapine versus switching to quetiapine in overweight or obese patients with schizophrenia or schizoaffective disorder. *Ther Clin Risk Manag*, 4(4), 713-20 (2008) doi:10.2147/tcrm.s3153

7. X. Fan, C. P. Borba, P. Copeland, D. Hayden, O. Freudenreich, D. C. Goff and D. C. Henderson: Metabolic effects of adjunctive aripiprazole in clozapine-treated patients with schizophrenia. *Acta Psychiatr Scand*, 127(3), 217-26 (2013) doi:10.1111/acps.12009

8. W. W. Fleischhacker, M. E. Heikkinen, J. P. Olié, W. Landsberg, P. Dewaele, R. D. McQuade, J. Y. Loze, D. Hennicken and W. Kerselaers: Effects of adjunctive treatment with aripiprazole on body weight and clinical efficacy in schizophrenia patients treated with clozapine: a randomized, double-blind, placebo-controlled trial. *Int J Neuropsychopharmacol*, 13(8), 1115-25 (2010) doi:10.1017/s1461145710000490

9. J. W. Newcomer, J. A. Campos, R. N. Marcus, C. Breder, R. M. Berman, W. Kerselaers, J. L'Italien G, M. Nys, W. H. Carson and R. D. McQuade: A multicenter, randomized, double-blind study of the effects of aripiprazole in overweight subjects with schizophrenia or schizoaffective disorder switched from olanzapine. *J Clin Psychiatry*, 69(7), 1046-56 (2008) doi:10.4088/jcp.v69n0702

10. T. S. Stroup, J. P. McEvoy, K. D. Ring, R. H. Hamer, L. M. LaVange, M. S. Swartz, R. A. Rosenheck, D. O. Perkins, A. M. Nussbaum and J. A. Lieberman: A randomized trial examining the effectiveness of switching from olanzapine, quetiapine, or risperidone to aripiprazole to reduce metabolic risk: comparison of antipsychotics for metabolic problems (CAMP). *Am J Psychiatry*, 168(9), 947-56 (2011) doi:10.1176/appi.ajp.2011.10111609

11. R. A. Wani, M. A. Dar, R. K. Chandel, Y. H. Rather, I. Haq, A. Hussain and A. A. Malla: Effects of switching from olanzapine to aripiprazole on the metabolic profiles of patients with schizophrenia and metabolic syndrome: a double-blind, randomized, open-label study. *Neuropsychiatr Dis Treat*, 11, 685-93 (2015) doi:10.2147/ndt.S80925

12. J. Zhao, X. Song, X. Ai, X. Gu, G. Huang, X. Li, L. Pang, M. Ding, S. Ding and L. Lv: Adjunctive Aripiprazole Treatment for Risperidone-Induced Hyperprolactinemia: An 8-Week Randomized, Open-Label, Comparative Clinical Trial. *PLoS One*, 10(10), e0139717 (2015) doi:10.1371/journal.pone.0139717

13. T. Baptista, N. Rangel, V. Fernández, E. Carrizo, Y. El Fakih, E. Uzcátegui, T. Galeazzi, M. A. Gutiérrez, M. Servigna, A. Dávila, M. Uzcátegui, A. Serrano, L. Connell, S. Beaulieu and E. A. de Baptista: Metformin as an adjunctive treatment to control body weight and metabolic dysfunction during olanzapine administration: a multicentric, double-blind, placebo-controlled trial. *Schizophr Res*, 93(1-3), 99-108 (2007) doi:10.1016/j.schres.2007.03.029

14. E. Carrizo, V. Fernández, L. Connell, I. Sandia, D. Prieto, J. Mogollón, D. Valbuena, I. Fernández, E. A. de Baptista and T. Baptista: Extended release metformin for metabolic control assistance during prolonged clozapine administration: a 14 week, double-blind, parallel group, placebo-controlled study. *Schizophr Res*, 113(1), 19-26 (2009) doi:10.1016/j.schres.2009.05.007

15. C. H. Chen, M. C. Huang, C. F. Kao, S. K. Lin, P. H. Kuo, C. C. Chiu and M. L. Lu: Effects of adjunctive metformin on metabolic traits in nondiabetic clozapine-treated patients with schizophrenia and the effect of metformin discontinuation on body weight: a 24-week, randomized, double-blind, placebo-controlled study. *J Clin Psychiatry*, 74(5), e424-30 (2013) doi:10.4088/JCP.12m08186

16. C. C. Chiu, M. L. Lu, M. C. Huang, P. Y. Chen, Y. K. Lin, S. K. Lin and C. H. Chen: Effects of Low Dose Metformin on Metabolic Traits in Clozapine-Treated Schizophrenia Patients: An Exploratory Twelve-Week Randomized, Double-Blind, Placebo-Controlled Study. *PLoS One*, 11(12), e0168347 (2016) doi:10.1371/journal.pone.0168347

17. L. F. Jarskog, R. M. Hamer, D. J. Catellier, D. D. Stewart, L. Lavange, N. Ray, L. H. Golden, J. A. Lieberman and T. S. Stroup: Metformin for weight loss and metabolic control in overweight outpatients with schizophrenia and schizoaffective disorder. *Am J Psychiatry*, 170(9), 1032-40 (2013) doi:10.1176/appi.ajp.2013.12010127

18. R. R. Wu, F. Y. Zhang, K. M. Gao, J. J. Ou, P. Shao, H. Jin, W. B. Guo, P. K. Chan and J. P. Zhao: Metformin treatment of antipsychotic-induced dyslipidemia: an analysis of two randomized, placebo-controlled trials. *Mol Psychiatry*, 21(11), 1537-1544 (2016) doi:10.1038/mp.2015.221

19. A. Amrami-Weizman, R. Maayan, I. Gil-Ad, A. Pashinian, C. Fuchs, M. Kotler and M. Poyurovsky: The effect of reboxetine co-administration with olanzapine on metabolic and endocrine profile in schizophrenia patients. *Psychopharmacology (Berl)*, 230(1), 23-7 (2013) doi:10.1007/s00213-013-3199-1

20. S. S. M. Assunção, S. I. Ruschel, L. d. C. R. Rosa, J. A. O. Campos, M. J. O. Alves, O. L. Bracco and M. S. d. Lima: Weight gain management in patients with schizophrenia during treatment with olanzapine in association with nizatidine. *Brazilian Journal of Psychiatry*, 28, 270-276 (2006)

21. M. P. Ball, K. R. Warren, S. Feldman, R. P. McMahon, D. L. Kelly and R. W. Buchanan: Placebo-controlled trial of atomoxetine for weight reduction in people with schizophrenia treated with clozapine or olanzapine. *Clin Schizophr Relat Psychoses*, 5(1), 17-25 (2011) doi:10.3371/csrp.5.1.3

22. T. Baptista, E. Uzcátegui, N. Rangel, Y. El Fakih, T. Galeazzi, S. Beaulieu and E. A. de Baptista: Metformin plus sibutramine for olanzapine-associated weight gain and metabolic dysfunction in schizophrenia: a 12-week double-blind, placebo-controlled pilot study. *Psychiatry Res*, 159(1-2), 250-3 (2008) doi:10.1016/j.psychres.2008.01.011

23. T. Baptista, N. Rangel, Y. El Fakih, E. Uzcátegui, T. Galeazzi, S. Beaulieu and E. Araujo de Baptista: Rosiglitazone in the assistance of metabolic control during olanzapine administration in schizophrenia: a pilot double-blind, placebo-controlled, 12-week trial. *Pharmacopsychiatry*, 42(1), 14-9 (2009) doi:10.1055/s-0028-1085438

24. C. P. Borba, X. Fan, P. M. Copeland, A. Paiva, O. Freudenreich and D. C. Henderson: Placebo-controlled pilot study of ramelteon for adiposity and lipids in patients with schizophrenia. *J Clin Psychopharmacol*, 31(5), 653-8 (2011) doi:10.1097/JCP.0b013e31822bb573

25. X. Fan, P. Copeland, S. Nawras, A. Harrington, O. Freudenreich, D. C. Goff and D. C. Henderson: Adjunctive telmisartan treatment on body metabolism in clozapine or olanzapine treated patients with schizophrenia: a randomized, double blind, placebo controlled trial. *Psychopharmacology (Berl)*, 236(6), 1949-1957 (2019) doi:10.1007/s00213-019-5181-z

26. A. Ghaderi, H. R. Banafshe, N. Mirhosseini, M. Moradi, M. A. Karimi, F. Mehrzad, F. Bahmani and Z. Asemi: Clinical and metabolic response to vitamin D plus probiotic in schizophrenia patients. *BMC Psychiatry*, 19(1), 77 (2019) doi:10.1186/s12888-019-2059-x

27. D. C. Henderson, P. M. Copeland, T. B. Daley, C. P. Borba, C. Cather, D. D. Nguyen, P. M. Louie, A. E. Evins, O. Freudenreich, D. Hayden and D. C. Goff: A double-blind, placebo-controlled trial of sibutramine for olanzapine-associated weight gain. *Am J Psychiatry*, 162(5), 954-62 (2005) doi:10.1176/appi.ajp.162.5.954

28. D. C. Henderson, X. Fan, P. M. Copeland, C. P. Borba, T. B. Daley, D. D. Nguyen, H. Zhang, D. Hayden, O. Freudenreich, C. Cather, A. E. Evins and D. C. Goff: A double-blind, placebo-controlled trial of sibutramine for clozapine-associated weight gain. *Acta Psychiatr Scand*, 115(2), 101-5 (2007) doi:10.1111/j.1600-0447.2006.00855.x

29. D. C. Henderson, X. Fan, B. Sharma, P. M. Copeland, C. P. Borba, R. Boxill, O. Freudenreich, C. Cather, A. Eden Evins and D. C. Goff: A double-blind, placebo-controlled trial of rosiglitazone for clozapine-induced glucose metabolism impairment in patients with schizophrenia. *Acta Psychiatr Scand*, 119(6), 457-65 (2009) doi:10.1111/j.1600-0447.2008.01325.x

30. J. A. Holka-Pokorska, R. Radzio, M. Jarema and A. Wichniak: [The stabilizing effect of dehydroepiandrosterone on clinical parameters of metabolic syndrome in patients with schizophrenia treated with olanzapine - a randomized, double-blind trial]. *Psychiatr Pol*, 49(2), 363-76 (2015) doi:10.12740/pp/30180

31. P. L. Ishøy, F. K. Knop, B. V. Broberg, N. Bak, U. B. Andersen, N. R. Jørgensen, J. J. Holst, B. Y. Glenthøj and B. H. Ebdrup: Effect of GLP-1 receptor agonist treatment on body weight in obese antipsychotic-treated patients with schizophrenia: a randomized, placebo-controlled trial. *Diabetes Obes Metab*, 19(2), 162-171 (2017) doi:10.1111/dom.12795

32. G. Joffe, P. Takala, E. Tchoukhine, H. Hakko, M. Raidma, H. Putkonen, M. Eronen and P. Räsänen: Orlistat in clozapine- or olanzapine-treated patients with overweight or obesity: a 16-week randomized, double-blind, placebo-controlled trial. *J Clin Psychiatry*, 69(5), 706-11 (2008) doi:10.4088/jcp.v69n0503

33. A. Krivoy, R. Onn, Y. Vilner, E. Hochman, S. Weizman, A. Paz, S. Hess, R. Sagy, S. Kimhi-Nesher, E. Kalter, T. Friedman, Z. Friedman, G. Bormant, S. Trommer, A. Valevski and A. Weizman: Vitamin D Supplementation in Chronic Schizophrenia Patients Treated with Clozapine: A Randomized, Double-Blind, Placebo-controlled Clinical Trial. *EBioMedicine*, 26, 138-145 (2017) doi:10.1016/j.ebiom.2017.11.027

34. J. R. Larsen, L. Vedtofte, M. S. L. Jakobsen, H. R. Jespersen, M. I. Jakobsen, C. K. Svensson, K. Koyuncu, O. Schjerning, P. S. Oturai, A. Kjaer, J. Nielsen, J. J. Holst, C. T. Ekstrøm, C. U. Correll, T. Vilsbøll and A. Fink-Jensen: Effect of Liraglutide Treatment on Prediabetes and Overweight or Obesity in Clozapine- or Olanzapine-Treated Patients With Schizophrenia Spectrum Disorder: A Randomized Clinical Trial. *JAMA Psychiatry*, 74(7), 719-728 (2017) doi:10.1001/jamapsychiatry.2017.1220

35. J. Li, X. Li, E. Liu, P. Copeland, O. Freudenreich, D. C. Goff, D. C. Henderson, X. Song and X. Fan: No effect of adjunctive, repeated dose intranasal insulin treatment on body metabolism in patients with schizophrenia. *Schizophr Res*, 146(1-3), 40-5 (2013) doi:10.1016/j.schres.2013.01.034

36. F. Liu, L. Xie, B. Zhang, Y. Ruan, Y. Zeng, X. Xu, J. Zhao and X. Fan: No Effect of Adjunctive Minocycline Treatment on Body Metabolism in Patients With Schizophrenia. *J Clin Psychopharmacol*, 38(2), 125-128 (2018) doi:10.1097/jcp.0000000000000841

37. M. L. Lu, H. Y. Lane, S. K. Lin, K. P. Chen and W. H. Chang: Adjunctive fluvoxamine inhibits clozapine-related weight gain and metabolic disturbances. *J Clin Psychiatry*, 65(6), 766-71 (2004) doi:10.4088/jcp.v65n0607

38. M. L. Lu, T. T. Chen, P. H. Kuo, C. C. Hsu and C. H. Chen: Effects of adjunctive fluvoxamine on metabolic parameters and psychopathology in clozapine-treated patients with schizophrenia: A 12-week, randomized, double-blind, placebo-controlled study. *Schizophr Res*, 193, 126-133 (2018) doi:10.1016/j.schres.2017.06.030

39. X. Lyu, J. Du, G. Zhan, Y. Wu, H. Su, Y. Zhu, F. Jarskog, M. Zhao and X. Fan: Naltrexone and Bupropion Combination Treatment for Smoking Cessation and Weight Loss in Patients With Schizophrenia. *Front Pharmacol*, 9, 181 (2018) doi:10.3389/fphar.2018.00181

40. A. Modabbernia, P. Heidari, R. Soleimani, A. Sobhani, Z. A. Roshan, S. Taslimi, M. Ashrafi and M. J. Modabbernia: Melatonin for prevention of metabolic side-effects of olanzapine in patients with first-episode schizophrenia: randomized double-blind placebo-controlled study. *J Psychiatr Res*, 53, 133-40 (2014) doi:10.1016/j.jpsychires.2014.02.013

41. F. Romo-Nava, D. Alvarez-Icaza González, A. Fresán-Orellana, R. Saracco Alvarez, C. Becerra-Palars, J. Moreno, M. P. Ontiveros Uribe, C. Berlanga, G. Heinze and R. M. Buijs: Melatonin attenuates antipsychotic metabolic effects: an eight-week randomized, double-blind, parallel-group, placebo-controlled clinical trial. *Bipolar Disord*, 16(4), 410-21 (2014) doi:10.1111/bdi.12196

42. R. C. Smith, H. Jin, C. Li, N. Bark, A. Shekhar, S. Dwivedi, C. Mortiere, J. Lohr, Q. Hu and J. M. Davis: Effects of pioglitazone on metabolic abnormalities, psychopathology, and cognitive function in schizophrenic patients treated with antipsychotic medication: a randomized double-blind study. *Schizophr Res*, 143(1), 18-24 (2013) doi:10.1016/j.schres.2012.10.023

43. F. Sun, Z. Ren, Y. Jiang, X. Fang, N. Wang and W. Jin: A Placebo-Controlled Study on the Treatment of Metabolic Syndrome of Qi Stagnation and Dampness Obstruction Related to Atypical Antipsychotics with Traditional Chinese Medicine (TCM). *Evid Based Complement Alternat Med*, 2020, 5103046 (2020) doi:10.1155/2020/5103046

44. E. Tavakoli, O. Rezaei and R. Fadai: A comparison on composition of celery, dill and green tea as three medicinal plants with placebo to treat schizophrenic patients' metabolic syndrome. *Afinidad*, 80(573), 1118-1123 (2014)

45. T. H. Taveira, W. C. Wu, E. Tschibelu, D. Borsook, D. C. Simonson, R. Yamamoto, D. D. Langleben, R. Swift and I. Elman: The effect of naltrexone on body fat mass in olanzapine-treated schizophrenic or schizoaffective patients: a randomized double-blind placebo-controlled pilot study. *J Psychopharmacol*, 28(4), 395-400 (2014) doi:10.1177/0269881113509904

46. C. Tek, J. Ratliff, E. Reutenauer, R. Ganguli and S. S. O'Malley: A randomized, double-blind, placebo-controlled pilot study of naltrexone to counteract antipsychotic-associated weight gain: proof of concept. *J Clin Psychopharmacol*, 34(5), 608-12 (2014) doi:10.1097/jcp.0000000000000192

47. L. Zhang, Y. Han, Z. Zhao, X. Liu, Y. Xu, G. Cui, X. Zhang and R. Zhang: Beneficial effects of konjac powder on lipid profile in schizophrenia with dyslipidemia: A randomized controlled trial. *Asia Pac J Clin Nutr*, 29(3), 505-512 (2020) doi:10.6133/apjcn.202009_29(3).0009

48. K. Zortea, V. C. Franco, L. P. Francesconi, K. M. Cereser, M. I. Lobato and P. S. Belmonte-de-Abreu: Resveratrol Supplementation in Schizophrenia Patients: A Randomized Clinical Trial Evaluating Serum Glucose and Cardiovascular Risk Factors. *Nutrients*, 8(2), 73 (2016) doi:10.3390/nu8020073
